# Chemical modulation of *Schistosoma mansoni* lysine specific demethylase 1 (SmLSD1) induces wide-scale biological and epigenomic changes

**DOI:** 10.12688/wellcomeopenres.18826.1

**Published:** 2023-03-30

**Authors:** Gilda Padalino, Cassandra A. Celatka, Hugh Y. Rienhoff Jr., Jay H. Kalin, Philip A. Cole, Damien Lassalle, Josephine Forde-Thomas, Iain W. Chalmers, Andrea Brancale, Christoph Grunau, Karl F. Hoffmann

**Affiliations:** 1 School of Pharmacy and Pharmaceutical Sciences, Cardiff University, Cardiff, Wales, CF10 3NB, UK; 2 Imago BioSciences, San Carlos, CA 94070, USA; 3 Division of Genetics, Department of Medicine, Brigham and Women’s Hospital and Department of Biological Chemistry and Molecular Pharmacology, Harvard Medical School, Boston, MA 02115, USA; 4 IHPE, University Perpignan Via Domitia, Perpignan, France; 5 Department of Life Sciences (DLS), Aberystwyth University, Aberystwyth, Wales, SY23 3DA, UK

**Keywords:** Lysine Specific Demethylase 1, epigenetics, ATAC-seq, anthelmintics

## Abstract

**Background**: *Schistosoma mansoni*, a parasitic worm species responsible for the neglected tropical disease schistosomiasis, undergoes strict developmental regulation of gene expression that is carefully controlled by both genetic and epigenetic processes. As inhibition of *S. mansoni* epigenetic machinery components impairs key transitions throughout the parasite’s digenetic lifecycle, a greater understanding of how epi-drugs affect molecular processes in schistosomes could lead to the development of new anthelmintics.

**Methods:** *In* *vitro* whole organism assays were used to assess the anti-schistosomal activity of 39 *Homo sapiens* Lysine Specific Demethylase 1 (HsLSD1) inhibitors on different parasite life cycle stages. Moreover, tissue-specific stains and genomic analysis shed light on the effect of these small molecules on the parasite biology.

**Results:** Amongst this collection of small molecules, compound **33** was the most potent in reducing *ex vivo* viabilities of schistosomula, juveniles, miracidia and adults. At its sub-lethal concentration to adults (3.13 µM), compound **33 **also significantly impacted oviposition, ovarian as well as vitellarian architecture and gonadal/neoblast stem cell proliferation. ATAC-seq analysis of adults demonstrated that compound **33** significantly affected chromatin structure (intragenic regions > intergenic regions), especially in genes differentially expressed in cell populations (e.g., germinal stem cells, hes2 *^+^* stem cell progeny, S1 cells and late female germinal cells) associated with these *ex vivo* phenotypes. KEGG analyses further highlighted that chromatin structure of genes associated with sugar metabolism as well as TGF-beta and Wnt signalling were also significantly perturbed by compound **33** treatment.

**Conclusions:** This work confirms the importance of histone methylation in *S. mansoni* lifecycle transitions, suggesting that evaluation of LSD1 - targeting epi-drugs may facilitate the search for next-generation anti-schistosomal drugs. The ability of compound **33** to modulate chromatin structure as well as inhibit parasite survival, oviposition and stem cell proliferation warrants further investigations of this compound and its epigenetic target SmLSD1.

## Introduction

Praziquantel (PZQ) is the only drug approved for the treatment of schistosomiasis, a neglected tropical disease (NTD) caused by infection with *Schistosoma* blood fluke parasites ^1, 2^. Due to obvious limitations of a mono-chemotherapeutic control strategy ^3– 6^, novel compounds with distinct mechanisms of action have been sought by researchers within both academic ^7– 9^ as well as industrial laboratories ^10^ for the development of alternative or combinatorial anti-schistosomals.

In this regard, two anti-schistosomal drug discovery strategies stand out. One involves the ‘re-purposing’ of approved drugs for new indications ^11, 12^. A second strategy involves the *de novo* design of drugs using either a ligand- or target-based molecular modelling approach ^13, 14^. Considering that epigenetic pathways play an important role in shaping schistosome phenotypes ^15^, controlling development ^16^ and responding to environmental stimuli ^17, 18^, pharmacologic inhibition of the key proteins by re-purposed or *de novo* designed compounds clearly defines a promising control strategy ^11, 15, 19, 20^.

Using both combinatorial chemistry and drug re-purposing, we and others have pursued the investigation of protein methylation components as next generation drug targets for schistosomiasis ^19– 22^ due to growing evidence of their impact on schistosome development and reproduction ^23^. Here, we further explore *S. mansoni* Lysine Specific Demethylase 1 (SmLSD1, Smp_150560) ^19, 21^ as a drug target using both late- and early-stage chemical entities developed to inhibit *Homo sapiens* LSD1 (HsLSD1) ^24– 28^.

Discovered in 2004, HsLSD1 is a histone H3K4/K9 mono- and di-methyl demethylase that employs flavin adenine dinucleotide (FAD) as a cofactor ^29^. LSD1 has been extensively explored as a drug target due to its regulatory activity as part of protein complexes involved in diverse biological processes ^28, 30^. Indeed, dysregulation of LSD1 function has been connected to pathophysiological conditions associated with diabetes, cancer, neurodegeneration and viral infection ^31, 32^. Therefore, the development of LSD1 therapeutics has been widely investigated ^33, 34^. Since the characterisation of the first LSD1 inhibitor, trans-2-phenylcyclopropylamine (2-PCPA, tranylcypromine), a large family of small molecule, mechanism-based, irreversible inhibitors that covalently modify FAD have been developed as therapeutic agents ^24, 35^. Additionally, peptide analogues of the histone H3 substrate have been identified as competitive LSD1 inhibitors ^25^. Other compounds have been identified that disrupt essential protein-protein interactions within the LSD1 complex or the binding of the LSD1-containing complex to the nucleosome ^36, 37^.

Therefore, to build upon recent successes in small molecule targeting of SmLSD1 ^19, 21^, we investigated the anthelmintic activity of a library of 39 HsLSD1 inhibitors. We reveal a critical role for SmLSD1 in miracidia to sporocyst transitioning, schistosomula/juvenile worm survival, adult worm motility and egg production. Furthermore, we show that small molecule targeting of SmLSD1 in adults reduces neoblast/gonadal stem cell proliferation, inhibits the formation of vitelline droplets in mature vitellocytes, affects ovarian architecture and induces wide-scale alterations in chromatin structure. Collectively, these data provide a molecular mechanism underpinning the parasite phenotypes and support further investigation of compounds inhibiting this histone-modifying enzyme in the pursuit of novel anti-schistosomals.

## Methods

### Ethics statement

All procedures performed on mice adhered to the United Kingdom Home Office Animals (Scientific Procedures) Act of 1986 (project licenses PPL 40/3700 and P3B8C46FD) as well as the European Union Animals Directive 2010/63/EU and were approved by Aberystwyth University’s Animal Welfare and Ethical Review Body (AWERB). All animals in this investigation were under the care of a Named Animal Care and Welfare Officer (NACWO), a Named Veterinary Surgeon (NVS), a small animal technician, a personal license holder (PIL) and a project license holder (PPL). While the procedure performed on these mice (infection with *S. mansoni* parasites) is classified within the moderate severity band of our license, efforts to ameliorate harm (outside of that induced by natural parasitic infection) included: provision of environmental enrichment stimulators (ameliorates mental harm), daily welfare and body condition checks (increasing to twice daily at day 45 post-infection) to avoid breaching the severity band of the project license and infection with a minimal number of parasites to reduce the likelihood of developing more than moderately adverse effects.

### Compound preparation and storage

All compounds were received as a dry powder. Upon delivery, each compound was dissolved in dimethyl sulfoxide (DMSO, 276855, Merck, UK) at a stock concentration of 10 mM and a working concentration of 1.6 mM (or lower, if needed). Both stock and working solutions were stored at -20°C prior to use. Positive controls for *S. mansoni* screens included praziquantel (PZQ, P4668, Sigma-Aldrich, UK) and auranofin (AUR, A6733, Sigma-Aldrich, UK); these were also solubilised in DMSO and stored as described above.

### Ligand preparation, protein preparation and molecular docking

From the chemical structure of compound **33** (an N-alkylated tranylcypromine derivative) and the cofactor FAD ( *Extended data,* Figure S7A and Figure S7B, respectively ^38^), the covalent adduct was generated using the builder tool in the software MOE (Molecular Operating Environment) 2015.10 ^39^. An open-access alternative that can perform an equivalent function is AutoDock ^40^. In brief, the chemical structure of FAD was firstly obtained from a selected crystal structure of HsLSD1 (PDB entry: 6NQU, 41 % sequence identity) and then its structure was simplified since only the flavin ring of the cofactor is involved in the mechanism of action of this class of LSD1 irreversible inhibitor. Thereafter, the desired ligand was obtained by forming a covalent adduct between the amino group of the cyclopropyl core of compound **33** and the flavin ring of the cofactor. As a result, the substitution on the amino moiety of this trans-2-phencylcyclopropylamine analogue (which most likely acts as lysine fragment mimic) is lost as shown in *Extended data* Figure S6C ^38^.

The obtained N5 adduct was saved in a *sdf* format prior to processing by the Lig Prep tool within Maestro v10.1 ^41^. An open-access alternative that can perform an equivalent function is AutoDock ^40^. For each ligand, 25 conformers of the ligand were generated and used for docking simulations. The homology model of Smp_150560 (SmLSD1) was generated within the MOE 2015.10 homology tool using a single template approach. Ten different intermediate models were built and minimised using Amber94 before refining the final model from a Cartesian average of the 10 generated intermediates ^19^. Similarly to ligand preparation, the structure of the cofactor (used for the induced-fit homology modelling of SmLSD1) was simplified. Here, only the tricyclic ring of FAD was kept whereas the 5´-adenosyldiphosphoribityl group at position 10 of the flavin ring (oriented towards the interior of the protein) was removed using the builder tool in MOE. The generated model was prepared with the Schrodinger Protein Preparation Wizard tool using the OPLS_2005 force field where hydrogens atoms were added, partial charges were assigned, and energy minimisation was performed. To facilitate docking, the flavin ring of FAD was selected as the centroid to computationally prepare a 12 Å docking grid. Docking simulations were performed using the Glide docking software and the in-built Extra Precision (XP) scoring function in order to estimate the target-compound binding affinity (as expressed as XP score). Selecting default parameters, only 10 output poses (conformations) for ligand conformer were generated in the final step. AutoDock could be alternatively used as docking program.

### Parasite maintenance and preparation

The NMRI (Naval Medical Research Institute) strain of *S. mansoni* was used to maintain the life cycle. The intra-mammalian developmental stages were generated by infecting *Mus musculus* (HsdOLa:TO - Tuck Ordinary; Envigo, UK); the intra-molluscan developmental stages were propagated through two *Biomphalaria glabrata* strains - the NMRI albino and pigmented outbred strains ^42^.

*S. mansoni* cercariae were obtained from infected *B. glabrata* snails after 1 h of incubation at 26°C under intensified lighting conditions. Cercariae were collected in falcon tubes and incubated on ice for at least 1 h prior to transformation. Cercariae were then repeatedly passed through a 10 ml serological pipette for at least 10 min, until no clumps of cercariae were present in the solution in order to mechanically transform them in schistosomula ^43^.

*S. mansoni* juvenile or adult worms were recovered by hepatic portal vein perfusion ^44^ of mice previously infected for 3 weeks with 4,000 cercariae/mouse or 7 weeks with 180 cercariae/mouse, respectively. Following perfusion, juvenile worms were collected by gravity in a 50 ml falcon tube, re-suspended in clear Dulbecco's Modified Eagle Medium (DMEM, 31053-028, Gibco, Paisley, UK) and washed three times (300 x *g* for 2 min) to remove residual host contamination. Adult worms were separated from perfusion media by sedimentation and subsequently washed a further three times in pre-warmed adult worm media (DMEM (Gibco, Paisley, UK) supplemented with 10% (v/v) Fetal Calf Serum (FCS, 31053-028, Gibco, Paisley, UK), 1% (v/v) L-glutamine (11539876, Gibco, Paisley, UK) and an antibiotic mixture (150 Units/ml penicillin and 150 µg/ml streptomycin; 15140122, Gibco, UK)). All parasite material was subsequently transferred into a petri dish and incubated in a humidified environment containing 5% CO _2_ at 37°C for at least one hour. Before downstream manipulation, any macro residual host material (e.g., mouse hair, blood clots) was removed using a clean paintbrush.

To obtain miracidia, parasite eggs were isolated from infected mouse livers (at 7 weeks post infection) by homogenisation of infected mouse livers in 2X saline solution using a Waring blender. The obtained eggs were exposed to light to induce miracidia hatching in 1X Lepple water ^23, 45^. Following hatching of miracidia, parasites were incubated on ice for 15 min and then centrifuged at 700 x *g* for 5 min at 4°C. The miracidia pellet was then re-suspended in 5 ml of Chernin's balanced salt solution (CBSS) ^23^, subjected to pelleting and two subsequent washes (all at 700 x *g* for 5 min at 4°C). Afterwards, the supernatant was carefully removed with a serological pipette and the miracidia-enriched pellet was resuspended with CBSS supplemented with 500 µl of penicillin-streptomycin (containing 10,000 units penicillin and 10 mg streptomycin/ml, P4333, Sigma-Aldrich, UK).

### *Ex vivo* schistosomula screens

Schistosomula compound screens were performed using an in-house facility, Roboworm, which quantifies both larva motility and phenotype metrics ^46^. Alternatively, the computer application Worminator can be used to assess motion of microscopic parasites such as the schistosomula stage of *S. mansoni* ^47^. Each compound (as single concentration or two-fold titration) was transferred with an automatic liquid handling platform into individual wells of a 384-well tissue culture plate (Perkin Elmer, cat 6007460) containing 20 µl of media. Following to that, the transformed schistosomula were dispensed in the plate at a specific seeding density (120 parasites in 60 µl, final volume) ^46, 48^. Each plate contained negative (0.625% DMSO) and positive (AUR at 10 µM final concentration in 0.625% DMSO) control wells. Schistosomula/compound co-cultures were then incubated at 37°C for 72 h in a humidified atmosphere containing 5% CO _2_. At 72 h, tissue culture plates were imaged under the same conditions (37°C for 72 h in a humidified atmosphere containing 5% CO _2_) using an ImageXpress Micro XL high content imager (Molecular Devices, UK) with subsequent images processed for phenotype and motility. Briefly, the motility was captured from five successive images taken at six second intervals, for the phenotype four adjacent images were taken and tiled together to maximise larval numbers for phenotype analysis. All images were taken by a high content imaging microscope (ImageXpress Micro XL) controlled with Meta Xpress software (Molecular Devices, UK) ^46^.

The phenotype and motility scores were used to evaluate whether a compound displayed anti-schistosomula activity; here, -0.15 and -0.35 defined threshold anti-schistosomula values for phenotype and motility scores, respectively. The Z´ value, a metric used to evaluate the success of high throughput -screens by comparing means and standard deviations of positive and negative controls ^49^, of each plate was subsequently calculated. Plates with a Z´ value below the value of 0.3 were considered failed and the screen was repeated.

### *Ex vivo* juvenile worm screens

Juvenile worms were distributed into a clear 96 well flat-bottom plate (25–30 juveniles/well, 200 µl of adult worm medium) and co-incubated with compounds (compound **33**: 20 – 0.63 µM in 1.25% DMSO; negative control: 1.25% DMSO; positive controls: 15 µM PZQ or 15 µM AUR in 1.25% DMSO; media only) ^48^. Treated juvenile worms were incubated for 72 h in a humidified environment containing 5% CO _2_ at 37°C. After 72 h, an adapted version of the World Health Organization Tropical Disease Research (WHO-TDR) scoring system ^50^ was used to quantify the effect of the compound on both phenotype and motility of the parasite ^48^. Juvenile worm viability was additionally quantified as previously reported with minor modifications ^51^. Briefly, propidium iodide (PI, P1304MP, Sigma-Aldrich, UK) was added to juvenile/compound co-cultures (to a final concentration of 2 µg/ml) and these co-cultures were imaged under both bright-field and fluorescent settings (PI detection, 535 and 617 excitation and emission wavelengths, respectively – using the Texas Red® channel Ex = 562/40nm, Em = 624/40nm), by an ImageXpress Micro XL high content imager (Molecular Devices, UK). All statistical analyses were performed using a two-way ANOVA followed by least significant difference post-hoc correction. All analysis were performed using GraphPad software, version 7 (RRID:SCR_002798).

### *Ex vivo* adult worm screens

Adult worms (1 worm pair/1 ml of adult worm media) were dosed with compounds at final concentrations spanning 50 µM – 0.78 µM (in 0.5% DMSO) in 48 well tissue culture plates. DMSO (0.5%) and praziquantel (10 µM in 0.5% DMSO) were also included as negative and positive control treatments. Treated adult worms were incubated for 72 h in a humidified environment at 5% CO _2, _37°C. Parasite motility after compound treatment was assessed by a digital image processing-based system (WormassayGP2) ^52, 53^ modified after Wormassay ^47, 54^.

Where egg deposition was noticed, eggs were removed and counted using a Sedgewick rafter. After counting, eggs were immediately transferred to a 1 ml microfuge tube and centrifuged at 200 x *g* for 2 min. With the eggs loosely pelleted at the bottom of the microfuge tube, the remaining media was carefully removed, and formalin (10% v/v formaldehyde, R3180000500, Fischer Scientific) was added to the egg pellet. After that, the eggs were stored for long term at 4°C. All statistical analyses were performed using a two-way ANOVA followed by least significant difference post-hoc correction. All analysis were performed using GraphPad software, version 7 (RRID:SCR_002798).

### *Ex vivo* miracidia screens

Miracidia (20–50 individuals in CBSS) were transferred to a 24-well tissue culture plate and dosed with a titration of compound **33** (50, 25, 10, 5, 2 and 0.5 µM in 1% DMSO) ^23^. Each treatment was performed in duplicate; parasites cultured in CBSS with 1% DMSO were included as a negative control. Miracidia were incubated for 48 h at 26°C and subsequently evaluated for morphological and behavioural changes differing from control cultures (1% DMSO) using an Olympus inverted light microscope. Dead, fully transformed and partially transformed miracidia were enumerated in the DMSO control and the compound cultures according the following criteria: dead if no sign of movement/flame-cell activity and extensive surface degradation was observed; fully transformed if the sporocyst surface was fully formed, no cilia plates remained attached and normal movement was detectable; partially transformed if the parasite presented a rounded morphology and ciliated plates were continuously shed ^12, 23, 55^. Miracidia were scored as fully transformed if the transformation process was completed, the sporocyst surface was fully formed, no cilia plates remained attached and normal movement was detectable. A miracidium was scored as partially transformed if the parasite was no longer swimming, assumed a rounded morphology and ciliated plates remained attached to the parasite surface. Dead parasites were identified if they did not show any signs of movement, extensive degradation was present at the surface and no flame-cell activity was evident. All statistical analyses were performed using a two-way ANOVA followed by least significant difference post-hoc correction. All analysis were performed using GraphPad software, version 7 (RRID:SCR_002798).

### Parasite staining and imaging

The polyphenol-rich vitelline droplets of mature vitellocytes were detected with Fast Blue BB Briefly, six worm pairs (derived from three independent replicates) were maintained in culture medium with or without 3.13 µM compound **33** (+ 0.5% DMSO) for 72 h. Afterwards, female worms were separated from males using 0.25% tricaine (886-86-2, Sigma-Aldrich) in DMEM, fixed in 4% formaldehyde (FX0415, Merck) in PBSTx (1X PBS supplemented with 0.3% Triton X-100 – X100, Merck) for 4 h and then washed in PBSTx for 10 min. A freshly made solution of 1% Fast Blue BB (L09704.06, Thermo Fisher Scientific) in PBSTx was used to stain the females for 5 min before being rinsed in PBSTx three times, cleared in 80% glycerol (G5516, Merck) in PBS and mounted on slides; Females were imaged using a Leica LMD6000 Laser Microdissection Microscope (10X objective) ^56, 57^.

To visualise the ultrastructural changes induced by compound **33**, staining of adult females with Langeron’s Carmine stain was employed ^58– 61^. Briefly, five worm pairs, maintained in either culture media (plus 0.5% DMSO) or 3.13 μM of compound **33** (+ 0.5% DMSO) for 72 h were killed with 0.6 M MgCl _2 _and fixed in AFA (2% glacial acetic acid - A6283, Merck, 25% formalin (i.e., 10% formaldehyde final), 48% ethanol (EtOH, 100983, Supelco) and 25% water) for at least 24 h. Next, the worms were washed in 70% EtOH (5 min) and stained in Langeron’s Carmine (340174N, Merck) for 30 min. Differentiation in acidic alcohol (2% HCl in 70% EtOH) followed until no more colour was released by the samples. Subsequently, the worms were dehydrated through a 70, 80, 90 and 100% EtOH series (2 min in each). Specimens were then cleared in xylene (Fisher Scientific) and whole worms were carefully transferred to pre-cleaned glass slides using a paintbrush and then mounted under a cover slip with DPX (06522, Merck). Confocal microscopy images were acquired using a Leica TCS SP5II laser scanning confocal microscope equipped with a 40X oil immersion objective and a 488 nm Argon laser (to detect autofluorescence, a scan excitation of 25%) and a 561 nm DPSS laser (to detect Carmine stain). Confocal Z-stacks (number of steps = 60, zoom factor = 1) were collected at 0.5 – 1 μm intervals to a total variable optical depth dependent on the thickness of the worm. The length of the ovaries was measured using ImageJ from the confocal Z-stacks described above ^62, 63^. All statistical analyses were performed using a nonparametric Student’s t-test (Mann-Whitney test). All analysis were performed using GraphPad software, version 7 (RRID:SCR_002798).

### Vitellocyte and egg volume quantification

The total number of *in vitro* laid eggs (IVLEs) produced by worms cultured in the presence of compound **33** (3.13 μM in 0.5% DMSO) or 0.5% DMSO were counted and subsequently fixed in 10% formaldehyde for at least 2 h at 4°C, or derived from long term storage in 10% formaldehyde at 4°C. Eggs were prepared for laser scanning confocal microscopy (LSCM) visualisation using DAPI (4’,6-diamidino-2-phenylindole, D9542, Merck) stain as previously described ^20^. Briefly, the eggs stored in formaldehyde were centrifugated, the excess solvent was removed and 2 μg/ml DAPI in PBS was added to the egg pellet at room temperature. Eggs were then carefully transferred to pre-cleaned glass slides for confocal microscopy. Fluorescent microscopic images (10 eggs per treatment) were acquired on a Leica TCS SP8 super resolution laser confocal microscope fitted with a 63X objective (water immersion, 1.75 zoom factor) using the Leica Application Suite X. For each Z-stack, a total of 60 sections were acquired selecting the 488 nm and 405 nm fluorescent channels for egg autofluorescence and DAPI (nuclei) stain, respectively. Quantification of overall volume (mapped by the green autofluorescence) and content of vitellocytes (DAPI) for individual eggs was performed using Imaris v8.2 (Bitplane, RRID:SCR_007370). All statistical analyses were performed using a nonparametric Student’s t-test (Mann-Whitney test). All analysis were performed using GraphPad software, version 7 (RRID:SCR_002798).

### Quantification of adult worm stem cell proliferation

*In vitro* 5′-ethynyl-2′-deoxyuridine (EdU) labelling of adult worms treated with compound **33** (3.13 µM in 0.5% DMSO) or 0.5% DMSO was performed as previously described ^64^. Briefly, adult worms were cultured for 48 h and pulsed with 10 µM EdU for the following 24 h. Following incubation, the worms were fixed with 4% formaldehyde (4 hours, at room temperature) and stained with detection solution (100 mM CuSO _4_, 10 mM AlexaFluor 488 conjugated Azide - A10266, Thermo Fischer, 0.5 M Ascorbic acid – 30 minutes at room temperature) and, after that, with DAPI (final concentration 5 µg/ml in PBS). The stained worms were then transferred on glass microscope slides using a paintbrush and then mounted under a cover slip with DPX (06522, Merck). Anterior, posterior and gonadal regions (ovaries for females and testes for males) of both sexes were imaged using a Leica TCS SP5II confocal microscope (40X objective, 1 zoom factor). A Z-stack, containing 60 sections, was generated for each microscopic image for each adult schistosome examined (six male and six female worms for each treatment). For the quantitative analysis, the fluorescent intensity of the DAPI (staining non-proliferating nuclei) and EdU (staining all dividing cells) channels were used to calculate the total volume (μm ^3^) occupied by each fluorophore using the Surface tool in Imaris v8.2 (Bitplane, RRID:SCR_007370). The percentage of EdU positive nuclei was calculated by dividing the volume of the EdU channel by the volume of the DAPI channel ^15^. All statistical analyses were performed using a nonparametric Student’s t-test (Mann-Whitney test). All analysis were performed using GraphPad software, version 7 (RRID:SCR_002798).

### ATAC-seq protocol

Individual worm pairs were cultured for 72 h in 3.13 µM of compound **33** (in 0.5% DMSO; n = 12 replicates) or in 0.5% DMSO (n = 12 replicates). After culture, individual worms were separated, transferred into Eppendorf tubes with forceps and washed once with cold PBS before the addition of 50 µl of transposase mixture (prepared as previously described ^65^). Briefly, the worm material was manually disrupted with disposable polypropylene pestles (Fisher, 10735925), the tagmentation reaction was carried out with lllumina Tagment DNA Enzyme and Buffer Kit (Illumina, 20034197, RRID:SCR_010233) and immediately purified (using QIAquick PCR Purification Kit,28104, Qiagen) following manufacturer’s instructions ^65^. 

Generation of ATAC-Seq libraries and bioinformatics analysis was done as described earlier ^65^. Briefly, universal index Ad1 and barcode Ad2 primers (as described in 65, sequence reported in *Extended data,* Table S8 ^66^) were used for each sample during library preparation/amplification. A preliminary 5-cycle amplification was carried out using NEBNext High-Fidelity 2X PCR Master Mix (M0541S, New England Biolabs) in a final volume of 50 μl. Following that, a qPCR side reaction was prepared with 5 μl PCR product of the initial pre-amplification reaction (keeping the remaining 45 µl at 4°C) along with 5 μl of SensiFAST™ SYBR® Hi-ROX Kit (BIO-92005, Scientific Lab Supplies), 4.5 μl of water, 0.25 μl of Universal Ad1_noMX primer (25 μM - same for all the 24 samples) and 0.25 μl of Specific Index primer - Ad2.* with * from 1 to 24 (25 μM - different for each sample). The optimal additional number of cycles needed for the remaining 45 μl PCR were calculated using the number of cycles that corresponded to 1/3 of the qPCR amplification curve slope during the exponential phase, as previously described ^65^. The number of additional cycles for this study is indicated in *Extended data,* Table S8 ^66^, column E. The resulting reaction was purified with QIAquick PCR Purification Kit (28104, Qiagen) and eluted into a total of 20 μl of elution buffer.

Illumina sequencing was performed on a Nextseq 550 version 4.0.1.41 (Illumina, USA) in paired-ends of 75 bp with two runs separated by a mid-flow cell (one with DMSO- and one with compound **33** treated-samples). The total number of sequencing reads for the libraries derived from the 24 compound **33**-treated individuals was 311,936,121 with an average of 12,997,338 reads/library. The total number of sequencing reads for the libraries derived from the 24 DMSO mock treated individuals was 332,262,454 with an average of 13,844,269 reads/library.

Quality check of reads was carried out with FastQC and summarised by MultiQC ^67^. Quality filtering and adapter removal was done with Trim Galore using default parameters. Alignment to the *S. mansoni* v7 genome was performed with Bowtie 2 ^68^ evoking the “very-sensitive end-to-end2” (‘—very-sensitive’) parameter. To identify regions in which ATAC-Seq reads were enriched, we decided to merge the BAM files generated by Bowtie for every single sample by condition and/or sex and to keep only the uniquely aligned reads using the Bowtie XS: tag. Local enrichments of aligned reads (peak calling) on (i) the merged uniquely aligned reads and (ii) all aligned reads for the individual sample libraries were done with MACS ^69^ callpeak, evoking the following parameters: genome size (‘gsize’) as 350,000,000, keep duplicate tags at the exact same location (‘keep-dup 1 –bdg’), *p*-value cut off (‘qvalue’) as 0.05, lower and higher model fold bound (‘mfold’) ^5, 50^, band width (‘bw’) as 1,500. Background correction was performed with MACS bdgcmp and the Poisson *p*-value method (‘-ppois’). To determine the number of aligned reads in the peak regions, BEDTools Intersect ^70^ intervals were obtained from the BED file of the peaks and the BAM alignment files of every single sample library without filtering the uniquely aligned reads. Annotation file schistosoma_mansoni.PRJEA36577.WBPS14.annotations.gff3 ^71^ was used to identify genomic features within genes. ChIPSeeker ^72^ allowed for association of the annotation files and ATAC-Seq peak regions. Using ChIPSeeker, 71,859 of the 71,892 ATAC-Seq peaks (>99%) and 84,998 of the 85,010 total peaks (~100%) were annotated in females and males, respectively. By default, in ChIPSeeker, the transcription start sites (TSS) region is defined from -3kb to +3kb. To produce metagene profiles, only the 5073 plus strand genes were filtered from the gff file. Compute Matrix of the DeepTools package ^73^ was used with the ‘scale-regions’ option, defining the region Body Length (distance in bases to which all regions are going to be fit) as 2,000 and up- and downstream distance of 2,000. PlotProfile of the same package served to produce images of metagene profiles evoking the “standard error” option. Significant differential peaks with adjusted *p*-value ≤ 0.05 were retained for the analysis. Integrative Genomics Viewer (IGV) was used for visualisation of MACS generated BedGraph files and BED files.

### Data visualisation

Heatmaps of enriched ATAC-Seq reads in significantly different peak regions were generated with Heatmapper. Three datasets were generated for each sex: (i) the raw number of reads aligned to each peak region that was also used as input to DESeq2, (ii) raw number divided by total number of peaks per sample and (iii) the normalised output of DESeq2. As expected, all files delivered roughly the same clustered heatmaps. Only differential peaks were shown here. Average linkage was used as the clustering method; Manhattan was used as distance Measurement Method; clustering was applied to rows and columns.

### KEGG enrichment analysis

To identify potential functions of genes or signalling pathways enriched (adjusted *p* value ≤ 0.05) in the ATAC-seq signals derived from the differential analyses (i.e., between DMSO and compound 33-treated), we conducted KEGG (Kyoto Encyclopedia of Genes and Genomes) enrichment analysis using ShinyGO (v. 0.76) ^74– 76^. The *p* < 0.05 value corrected using the Benjamini & Hochberg algorithm was set as the threshold for identifying the pathways.

### Western blot analysis

Following 72 h post treatment with a sublethal concentration of compound **33** (3.13 µM), adult male worms (n = 20 worms, three biological replicates) were homogenized with a TissueLyser (Qiagen) and total histones were extracted using the EpiQuikTM Total Histone Extraction kit (OP-0006, Epigentek) following the manufacturer’s instructions. A total of 2.5–10 µg of each sample was separated by sodium dodecyl sulfate-polyacrylamide gel electrophoresis (SDS-PAGE) through 12% Mini-PROTEAN® TGX™ Precast Gels (4561043, Biorad). After transferring the proteins onto a 0.2 µm Nitrocellulose membrane (1620150, Biorad) using a Trans-blot Turbo Midi system (Bio-Rad; Trans-blot turbo protocol - 25V and 2.5A during 3 minutes), the membranes were blocked overnight in 5% fat-free dry milk in Tris-buffered saline (TBS) supplemented with 0.05% Tween 20 (TBST). Following that, the membranes were probed with 1:2000 dilution of anti-H3K4me2 (ab32356, Lot GR84714-4, Abcam, RRID:AB_732924) overnight (at 4°C) in 5% fat-free dry milk in TBST. The blot was washed, incubated overnight at 4°C with 1:500 dilution of the secondary antibody (goat anti-rabbit Horseradish Peroxidase coupled antibody - Pierce #31460, Lot HB987318, RRID:AB_228341) in 5% fat-free dry milk in TBST. The blot was then developed by incubating with an enhanced chemiluminescence (ECL) substrate (Pierce) followed by CCD camera image acquisition. Acquisition time was adjusted to have maximum exposure without saturation. The detected bands were analysed with ChemiDoc software v4.0.1 with high sensitivity settings.

For sample normalisation, the membranes were stripped by incubation at 50°C for 1 h in 62 mM TRIS-HCl pH 6.8 (15435919, Fisher Scientific), 2% SDS (151-21-3, Melford), 0.8% Beta-Mercaptoethanol (M6250, Merck), then washed with distilled water twice for 5 min. The membranes were subsequently probed overnight at 4°C with an anti-H3 Ab (Abcam, ab1791, Lot GR103803-1), 1:1000 diluted in 5% non-fat dried milk in TBST. Detection of H3 signal by secondary antibody, image acquisition and analysis were all performed as described above.

### Hypergeometric analysis

Statistical analysis for enrichment of target genes within the 68 clusters was performed in Microsoft Excel 2021 (template saved in *Extended data,* Table S9 ^66^), using a hypergeometric test for overrepresentation ^77– 79^. The hypergeometric test, also called hypergeometric distribution, is a probability distribution describing the number of successes, selected from a population with no replacement.

## Results

### HsLSD1 inhibitors differentially affect schistosomula

**Figure 1.**
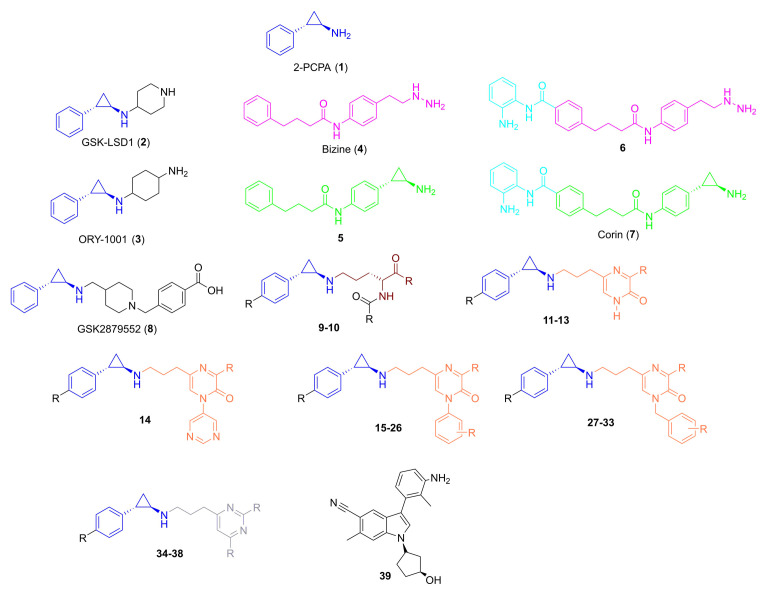
Nomenclature of LSD1 inhibitors assessed as potential anti-schistosomals. The chemical structures of tranylcypromine (2-PCPA, **1**), the first known LSD1 inhibitor, and other derived compounds developed as covalent inhibitors of LSD1 are shown. The compounds (numbered from **1** to **39**) are grouped in subclasses based on their structural similarity and a coloured code scheme is used to highlight common structural scaffolds: blue represents the phenyl substituted tranylcypromine core; cyan indicates the incorporated features of the HDAC inhibitor (Entinostat) coupled to either the established LSD1 inhibitor bizine (in magenta) or the cyclopropylamine analogue of bizine (compound **5**, in green); brown represents the lysine mimetic scaffold; orange denotes the propylpyrazin-2-(1H)-one alone or differently substituted with pyrimidine, phenyl or benzyl cores; light blue signifies the propyl-pyrimidine scaffold. The chemical structures of each compound are reported in *Extended data,* Table S1 ^66^. The commercial name of some compounds is also reported if known.

A small library of LSD1 inhibitors was acquired through commercial/collaborative sources and from information derived from a recent large-scale RNAi investigation of *S. mansoni* gene function ^53^ ( Figure 1 and *Extended data,* Table S1 ^66^). This collection included the only FDA-approved LSD1 inhibitor, trans-2-Phenylcyclopropylamine, (2-PCPA or better known as tranylcypromine ( **1**) ^35^), several small molecules undergoing clinical testing (including GSK-LSD1 ( **2**) ^80^, ORY-1001 ( **3**) ^81^ and GSK2879552 ( **8**) ^82^), pharmacologically active compounds synthesised around a substituted lysine scaffold (compounds **9** and **10** ^83, 84^, the latter currently in clinical trial for the treatment of myelofibrosis ^85^) and a selection of derivatised chemicals (compounds **11**– **38**) ^27, 86^. This collection also included two dual LSD1/HDAC (histone deacetylase) inhibitors (compounds **6** and **7**) ^26^ designed as hybrid compounds resulting from the combination of a standard HDAC zinc binding group (benzamide group of Entinostat (MS-275) ^87^) to either a phenelzine derivative ( **4**, also known as Bizine ^88^) or a cyclopropylamine analog of Bizine ( **5**, 88), respectively. This small library of LSD1 inhibitors also included a cyano-substituted indole compound (compound **39**) developed by Novartis for the treatment of LSD1-mediated diseases or disorders ^89^.

**Figure 2.**
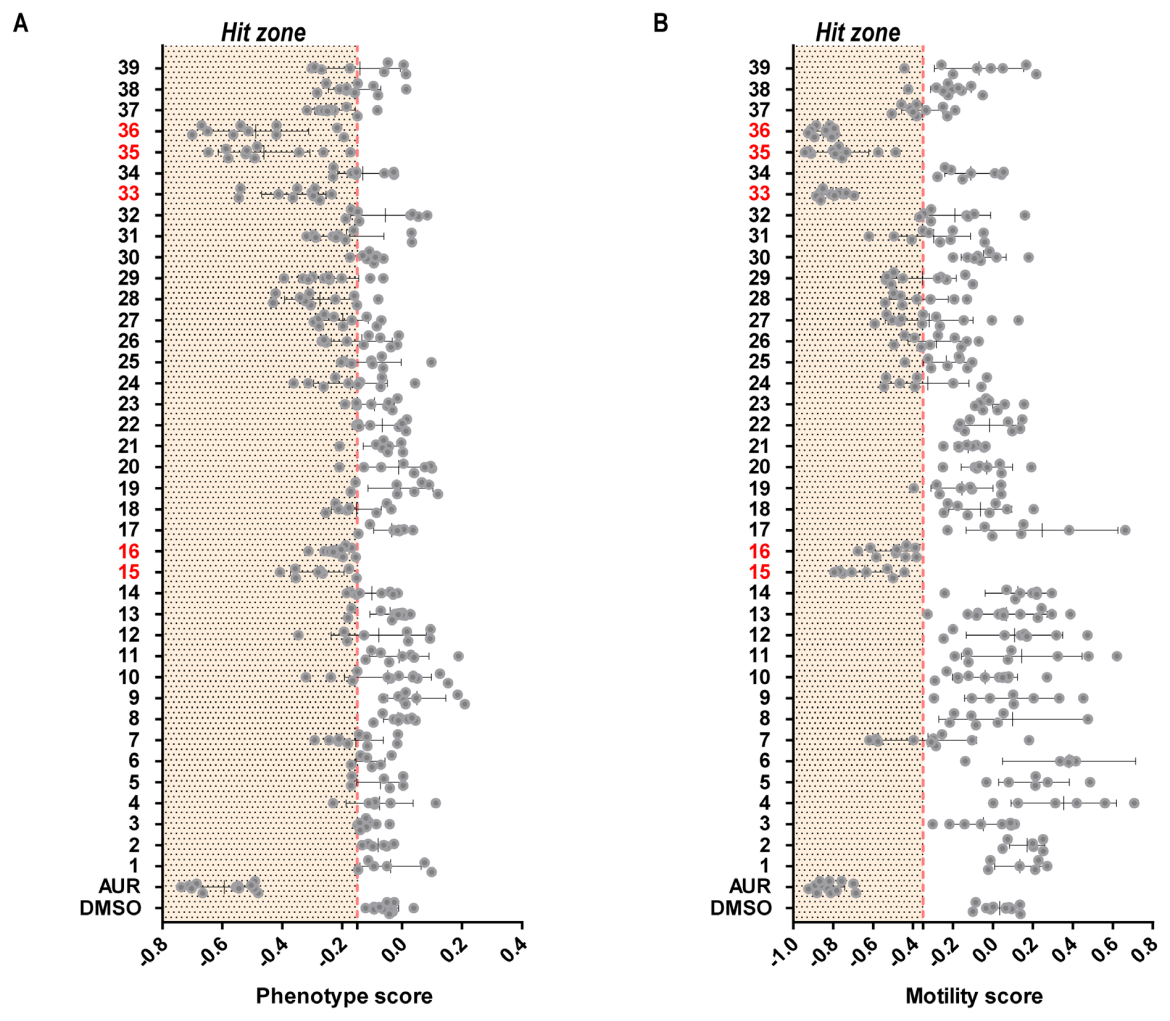
*Ex vivo* schistosomula screen of putative SmLSD1 inhibitors. Mechanically-transformed schistosomula (n = 120) were incubated with each of the 39 compounds for 72 h at 37°C in a humidified atmosphere containing 5% CO _2_. At 72 h, the effect that each compound had on parasite phenotype ( **A**) and motility ( **B**) was assessed by the high throughput platform Roboworm and compared to negative (0.625% DMSO) as well as positive (10 µM Auranofin in 0.625% DMSO) controls. Each compound was screened two to three times in three independent screens. The compound score is shown as grey dots and whiskers representing the average score and standard deviation across the screens. Compounds with activity on schistosomula phenotype and motility are shown within the ‘Hit Zone’ (delineated by the vertical dashed red lines in the graphs; - 0.15 and - 0.35 for phenotype and motility scores, respectively). All compounds showing a score lower than both reference values are considered hits (placed in an area highlighted as “Hit zone”). The five compounds highlighted in red were consistent hits across the independent screens. Z´ scores of drug screens are reported in *Extended data,* Table S2 ^66^.

The selected compounds were initially co-cultured with schistosomula for 72 h (10 µM final concentration). At this concentration, each compound was screened at least three times (biological replicates) and, in each screen, the effect of the compounds on schistosomula phenotype and motility was assessed twice (technical replicates) using the high-throughput Roboworm platform ^20, 46, 48^. For each screen, the calculated Z´ scores for both phenotype and motility metrics were within acceptable ranges ( *Extended data,* Table S2 ^66^) as previously described ^49^. Upon screening ( Figure 2), five compounds ( **15**, **16**, **33**, **35** and **36**, in red) were reproducibly identified as hits on both metrics (phenotype Figure 2A and motility Figure 2B) when compared to negative (0.625% DMSO) and positive (10 µM Auranofin - AUR in 0.625% DMSO) controls ^90^.

 In line with previous studies ^20^, GSK-LSD1 (compound **2**) failed to affect either schistosomula motility or phenotype. Amongst the five hits, compounds **33**, **35** and **36** appeared to be more potent than compounds **15** and **16** (i.e., schistosomula motility and phenotype scores for the first three compounds were lower than the latter two, Figure 2). A subsequent dose-response titration of these five compounds confirmed this observation (Z´ scores for both phenotype and motility metrics of each screen summarised in *Extended data,* Table S3 ^66^); EC _50_ (half maximal effective concentration) values for schistosomula phenotype metrics were higher for compounds **15** and **16** (9.50 and 7.57 µM, respectively) when compared to the remaining three (4.37, 5.03 and 4.72 µM for compounds **33**, **35** and **36**, *Extended data,* Figure S1 ^38^).

### Five HsLSD1 inhibitors affect adult worm motility and egg production

**Figure 3.**
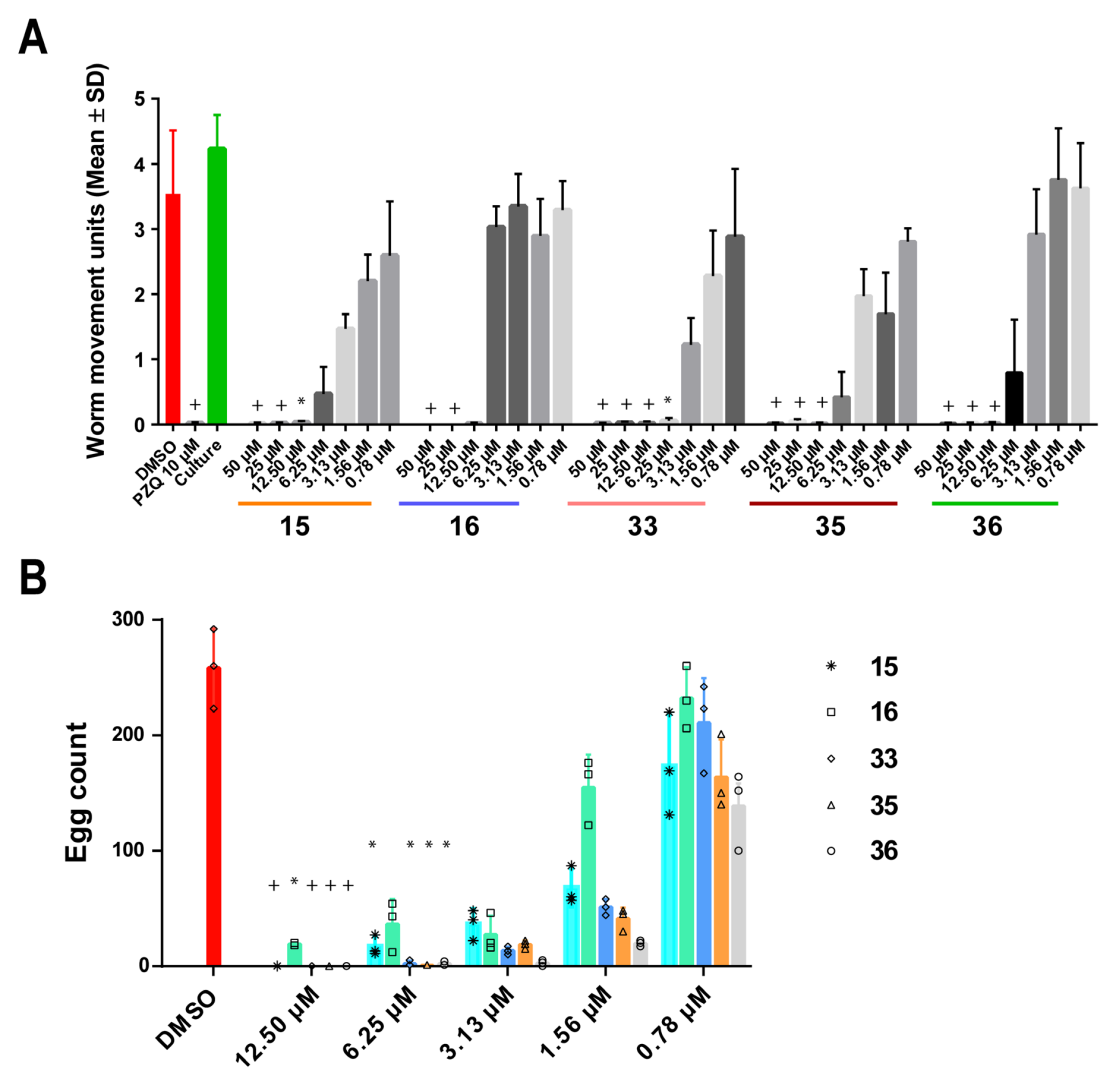
Adult worm motility and *in vitro* laid egg (IVLE) production are impaired by LSD1 inhibitors. A dose response titration (50 – 0.78 µM in 0.625% DMSO) of the five compounds with anti-schistosomula activity was performed to assess their potency on adult worms (1 pair/well; n = 6). The titration was performed in duplicate in three independent screens. ( **A**) - Worm movement was recorded at 72 h with WormassayGP2. The average worm movement (+ SD) of the three independent screens is indicated. Compound-inhibited worm movement is compared to controls (media containing 0.5% DMSO, culture control lacking 0.5% DMSO and 10 µM PZQ containing 0.5% DMSO). ( **B**) - At 72 h, eggs were collected and enumerated. For each concentration tested, individual egg counts are represented in a scatter plot; the average and standard error across the replicates is represented as a bar chart. A Kruskal-Wallis ANOVA followed by Dunn’s multiple comparisons test was performed to compare each population mean to DMSO mean. For both panels, * and + represent *p* < 0.0332 and *p* < 0.0021, respectively.

The effect of anti-schistosomula compounds ( **15**, **16**, **33**, **35** and **36**) on adult worm pairs (7 weeks old) was next explored to expand their anti-schistosomal applicability ( Figure 3). Here, all compounds had a lethal effect (i.e., absence of parasite motility and gut peristalsis for 30 seconds associated with parasite detachment from the tissue culture well) on the parasite at the highest concentrations tested (50 and 25 µM, Figure 3A). Similar findings were recorded for all compounds at 12.50 µM; the only exception being compound **16**, which severely inhibited parasite motility, but was not lethal. Upon further adult worm titrations, and consistent with the schistosomula screens, compound **33** displayed the greatest activity and inhibited schistosome motility at concentrations as low as 6.25 µM. At 3.13 μM, the effect of all compounds was minimal except for compound **33** ( *Extended data,* Movie S1 ^66^); at lower concentrations, the treated worms started recovering within the 72 h treatment window when compared to the control ( Figure 3A).

*In vitro* laid egg (IVLE) production was also affected by co-cultivation with each of the five compounds ( Figure 3B). Unsurprisingly, based on motility readouts ( Figure 3A), no eggs were recovered from the culture media after 72 h incubation with the highest concentrations (50 and 25 µM) of all five compounds. At 12.50 µM, few eggs were recovered after co-incubation with compound **16**. However, compounds **33**, **35** and **36** again demonstrated the strongest effects in inhibiting this critical process involved in host immunopathology and lifecycle transmission. For compounds **35** and **36**, inhibition of IVLE production persisted even at concentrations (i.e., 6.25 µM) where worm motility recovered. In addition to the reduced egg production, a number of free (not packaged in fully formed eggs) vitelline cells, oocytes and spermatozoa were observed in the culture medium following treatment with a sublethal dose of compound **33** (3.13 μM, *Extended data,* Figure S2 ^38^ and Movie S1 ^66^); this phenotype has previously been reported in a limited number of unrelated drug studies ^91– 93^. 

### Compound 33 reduces juvenile worm viability and miracidia transformation

**Figure 4.**
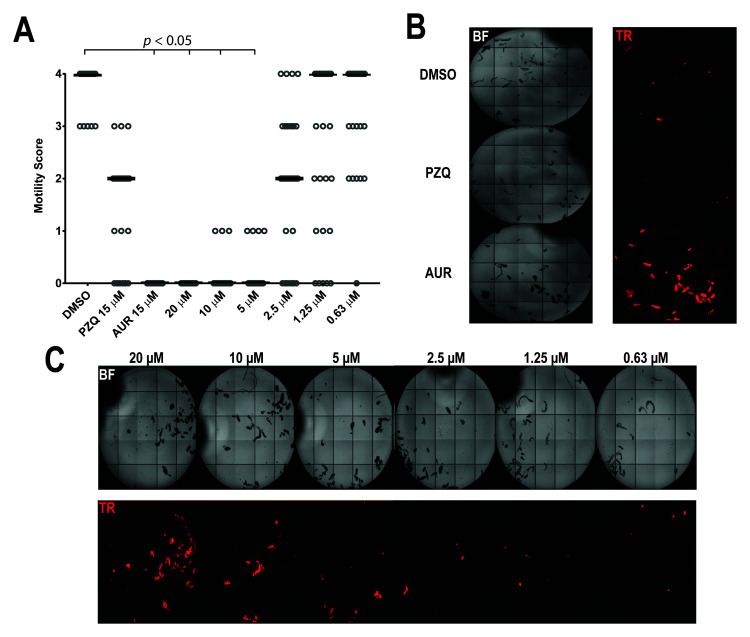
SmLSD1 inhibition leads to decreased juvenile worm motility and viability. Juvenile *S. mansoni* worms (3 weeks post infection; n = 25-30 parasites) were subjected to a dose response titration of compound **33** (20, 10, 5, 2.50, 1.25 µM and 0.63 µM in 1.25% DMSO). Motility (0 = no movement, 1 = movement of the suckers only and slight contraction of the body, 2 = movement of the anterior and posterior regions only, 3 = full body movement but sluggish, 4 = normal movement) and viability metrics (PI positive parasites) were assessed at 72 h post-dosing and compared to control parasites (negative control: 25–30 juveniles co-cultivated in the presence of 1.25% DMSO; positive controls: 25–30 juveniles co-cultured in either 15 µM PZQ or AUR in 1.25% DMSO). ( **A**) - The scatter plot shows the motility score for each parasite/treatment and the median value is indicated as a horizontal line. For statistical analysis of the median values, the Fisher’s exact test was performed in R studio. ( **B**) - Representative images of PI-stained (2 µg/ml) juveniles treated with DMSO, praziquantel (PZQ, 15 µM) and auranofin (AUR, 15 µM). ( **C**) - Representative images of compound **33**/parasite co-cultures showing a concentration-dependent increase in PI staining. The plate was imaged under both bright-field (BF) and fluorescent (Ex = 562/40nm, Em = 624/40nm, for PI detection) settings, using an ImageXpress Micro XL high content imager (Molecular Devices, UK).

The activity of compound **33** was further explored on two other important *S. mansoni* life cycle stages, the immature juvenile worms and the snail-infective miracidia. Firstly, juvenile (3 weeks old) schistosomes were subjected to dose response titrations of this compound for 72 h, after which both parasite motility and viability was assessed ( Figure 4).

At the highest concentration tested (20 µM), compound **33** significantly reduced parasite movement when compared to the negative (DMSO) control ( Figure 4A). A similar observation was recorded for auranofin (AUR)-treated parasites (at 15 µM). Furthermore, when visualised for propidium iodide (PI) uptake ( Figure 4B and Figure 4C), both treatments were associated with increased fluorescence, providing confirmation of juvenile worm death. In line with other reports ^6, 94, 95^, PZQ only showed partial activity on juvenile worms ( Figure 4A), and as confirmed by a lower uptake of PI ( Figure 4B), these parasites were not all dead. At lower concentrations of compound **33** (10, 5 and 2.50 µM), reductions in both motility ( Figure 4A) and viability ( Figure 4C) were still noted when compared to the DMSO control. However, at the lowest two concentrations (1.25 µM and 0.63 µM), juveniles started recovering normal movement within the 72 h treatment window and mortality was substantially reduced.

**Figure 5.**
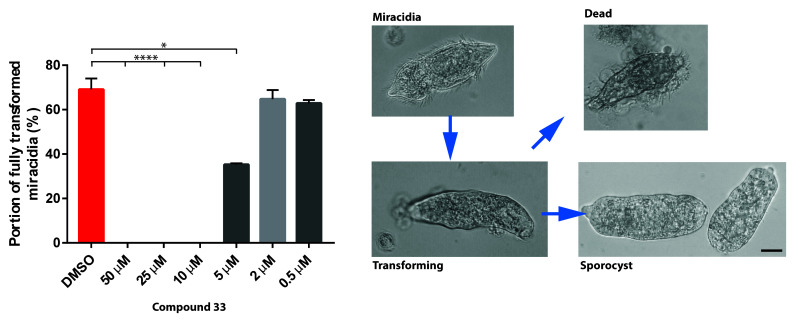
SmLSD1 inhibition blocks miracidia to sporocyst transformation. Miracidia were exposed to compound **33** during a dose response titration (50, 25, 10, 5, 2 and 0.5 µM in 1% DMSO). Sporocyst transformation was scored (%) after 48 h. Representative bright field images of miracidia, transforming sporocysts, fully-transformed sporocysts and dead miracidia/sporocysts are illustrated. Each titration point was conducted in triplicate and compared to parasites cultured in Chernin's balanced salt solution (CBSS) with 1% DMSO (controls) at a constant temperature of 26°C (in the dark). Means and standard errors are shown. Bar = 40 µm. A Kruskal-Wallis ANOVA followed by Dunn’s multiple comparisons test was performed to compare each population mean to DMSO mean. * and **** represent *p* < 0.0332 and *p* < 0.0001, respectively.

Although phenotypic screens were mainly focused on intra-mammalian parasitic stages, we were also interested in whether the most potent LSD1 inhibitor affected schistosome developmental forms that interact with the intermediate molluscan host ^55^. Hence, free-swimming miracidia were exposed to compound **33** (in a dose response titration) for 48 h and *in vitro* transformation into sporocysts was assessed ( Figure 5).

Upon titration, a significant inhibition in miracidia to sporocyst transformation was found for parasites treated with 50 – 5 µM of compound **33**. In fact, no movement or flame cell activity (related to parasite death) was observed at 50, 25 and 10 µM concentrations. At 5 µM, compound **33** inhibited miracidia-sporocyst transformation by 66%. Below 5 µM, miracidia to sporocyst transformation was unaffected.

### Compound 33 affects the female reproductive system and inhibits vitellocyte packaging

**Figure 6.**
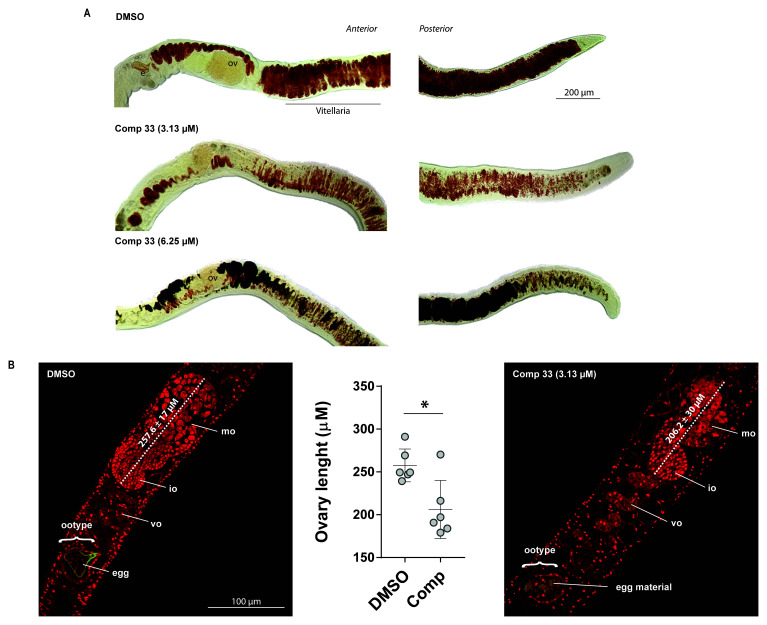
SmLSD1 inhibition significantly impacts both schistosome vitellarian and ovarian architecture. Adult schistosome pairs (1 pair/well; n = 6) were co-cultured for 72 h with a sub-lethal concentration of compound **33** (3.13 μM in 0.5% DMSO) or DMSO (0.5%). ( **A**) - Fast Blue BB staining (orange-red labelling) of vitelline droplets in mature vitellocytes in compound **33** - treated female worms compared to controls (DMSO). Representative images are taken from three experiments with n = 6 parasites. ‘e’ and ‘ov’ define egg and ovary, respectively. ( **B**) - Confocal laser scanning microscope images of adult females stained with Langeron’s Carmine. Representative images from two experiments with n = 6 parasites. Immature oocytes (io), mature oocytes (mo), vitello-oviduct (vo) and ootype are indicated. Average ovary length is given as the mean ± SD of six females across three experiments. Bar chart shows the ovary length of each individual worm. A Mann-Whitney test was performed to identify statistical difference between the treatments (* indicating *p* < 0.05).

As schistosomiasis immunopathology as well as parasite transmission are both directly related to egg production from sexually-mature adult female worms and compound **33** specifically inhibited this phenotype in *ex vivo* cultivated adults ( Figure 3), we next conducted a series of cell and molecular biological investigations to understand how this epi-drug exerts its activity on adult worm processes underlying oviposition. Firstly, the vitellaria of paired females cultured with sub-lethal concentrations of compound **33** (3.13 µM, which did not significantly reduce worm motility - Figure 3A) were stained with Fast Blue BB, a dye that specifically stains vitelline droplets in mature vitellocytes ( Figure 6A) ^56, 57^. Whereas Fast Blue BB ^+^ vitelline droplets (colour orange-red in appearance) and eggs containing lateral spine were present in the control worms, the vitellaria ceased production of large numbers of Fast Blue BB ^+^ mature vitellocytes (S4 cells) in compound-treated females; this effect was further enhanced in females treated with 6.25 µM compound **33**. No eggs were observed in either compound-treated females.

Secondly, compound **33** treatment also induced morphological changes in the ovary as observed upon carmine red staining ^58, 60, 61^. The control adult females (DMSO treated) showed immature oocytes (io or germinal stem cells, GSCs) in the anterior part and larger mature oocytes (mo) in the posterior part of their ovaries. For comparison, in all the compound treated females (6/6), a reduced number of mature oocytes was observed in more compacted (shrunken) ovaries. Although subtle, a significant decrease in ovary size (reduced length) was seen in treated worms (n = 6) compared with control worms (n = 6). Additionally, the ootype was found to be empty or full of egg material in all the compound treated females, whereas a fully mature egg was observed in the control female worms ( Figure 6B).

**Figure 7.**
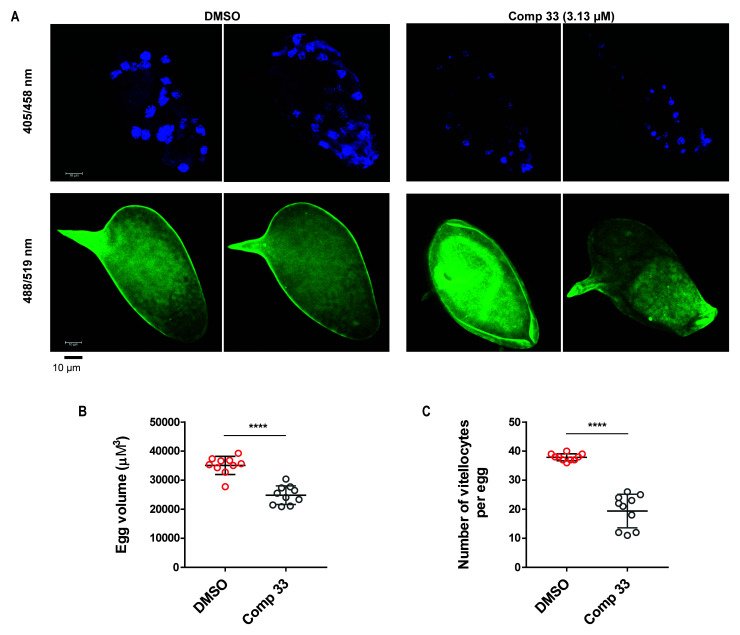
SmLSD1 inhibition significantly affects IVLE volume and vitellocyte packaging. ( **A**) Representative IVLE phenotypes (DAPI; Ex = 405 nm, Em = 458 nm and autofluorescence; Ex = 488 nm, Em = 519 nm) of eggs derived from compound **33** - treated worm cultures compared to the negative control cultures (0.5% DMSO). ( **B**) - Quantification of egg volumes between treatment (compound **33**) and DMSO control groups (n = 10 per group). ( **C**) - Number of vitellocytes per egg between treatment (compound **33**) and DMSO control groups (n = 10 per group). DAPI stain = blue; Autofluorescence = green. Mean and standard error are represented in Panels B (volume) and C (vitellocyte numbers). A Mann-Whitney test was subsequently performed to identify statistical difference between the treatments (**** corresponds to *p* < 0.0001).

Thirdly, eggs derived from schistosome cultures co-incubated with sub-lethal concentrations of compound **33** (3.13 µM) were analysed using confocal microscopy and compared to IVLEs derived from DMSO-treated worms ( Figure 7). Even though there were no evident phenotypic abnormalities (lateral spine and oval shape were both present) in the small number of IVLEs produced ( Figure 7A), a significant difference in overall egg volume was observed in compound **33** treated worms ( Figure 7B). Moreover, following vitellocyte quantification, this compound also significantly inhibited the number and packaging of this critical cell population into IVLEs ( Figure 7C and *Extended data,* Movie S2 ^66^) ^96, 97^.

### Compound 33 inhibits adult worm stem cell proliferation

**Figure 8.**
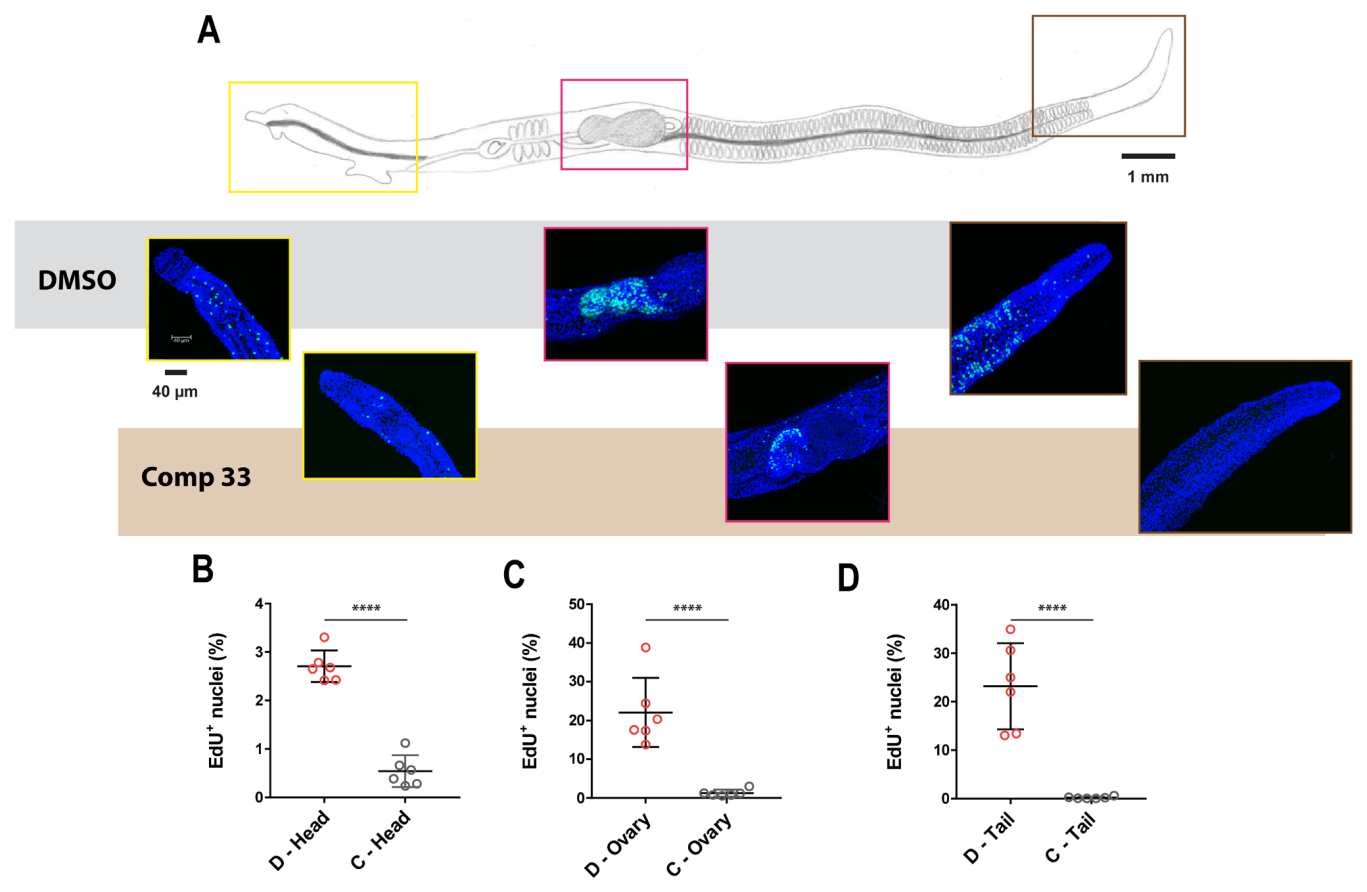
SmLSD1 inhibition reduces adult female stem cell proliferation. Female schistosomes treated with 3.13 μM of compound **33** (n = 6) or DMSO controls (n = 6) for 48 h were subsequently labelled with EdU for an additional 24 h. ( **A**) - Schematic of a *S. mansoni* adult female with representative anterior (yellow box), gonadal (magenta box) and posterior region (brown box) of untreated (top row, grey) compared to compound **33** - treated (bottom row, light brown) worms. Fluorescent microscopic images (six females per treatment) were acquired on a Leica TCS SP8 super resolution laser confocal microscope fitted with a 40X objective (water immersion, 1,00 zoom factor, Z stack of 60 steps) using the Leica Application Suite X. DAPI stain = blue; EdU+ cells = green. Scale bar represents either 1 mm or 40 μm. Scatter plots illustrate the percentage of proliferating cells present in control (D; DMSO treated worms, n = 6) versus compound **33** - treated females (C; 3.13 µM of compound **33**, n = 6) in the head region ( **B**), the ovary ( **C**) and the tail region ( **D**). Standard errors are shown, and a Mann-Whitney test was subsequently performed (with **** corresponding to *p* < 0.0001).

Due to a previous role ascribed for LSD1 in maintaining mammalian stem cell function ^98^, 5-ethynyl-2′-deoxyuridine (EdU) labelling of compound **33** treated adult worms was performed to assess neoblast and gonadal stem cell proliferation. EdU labelling was performed on both female ( Figure 8 and *Extended data,* Movie S3 ^66^) and male ( *Extended data,* Figure S4 ^38^ and Movie S4 ^66^) worms treated with a sublethal concentration (3.13 µM) of compound **33** for three days.

For each worm (n = 6), three different anatomical regions (anterior region - yellow, gonadal system - magenta and posterior region - brown) were observed, and the number of EdU ^+^ dividing cells was quantified (as a percentage of total DAPI ^+^ cells in the considered region). As shown in representative images of the parasite samples, a reduction in EdU ^+^ cells was detected in compound-treated adults when compared to controls (both female and male parasites, Figure 8 and *Extended data,* Figure S4 ^38^, respectively). Quantification of EdU ^+^ nuclei revealed that compound **33** reduced cellular proliferation similarly across the different anatomic regions of the parasite body ( Figure 8A and *Extended data,* Figure S4A ^38^), regardless of sex or stem cell source ( Figure 8 and *Extended data,* Figure S4 ^38^, panels **B–D**).

### Compound 33 disrupts chromatin structure in adults

**Figure 9.**
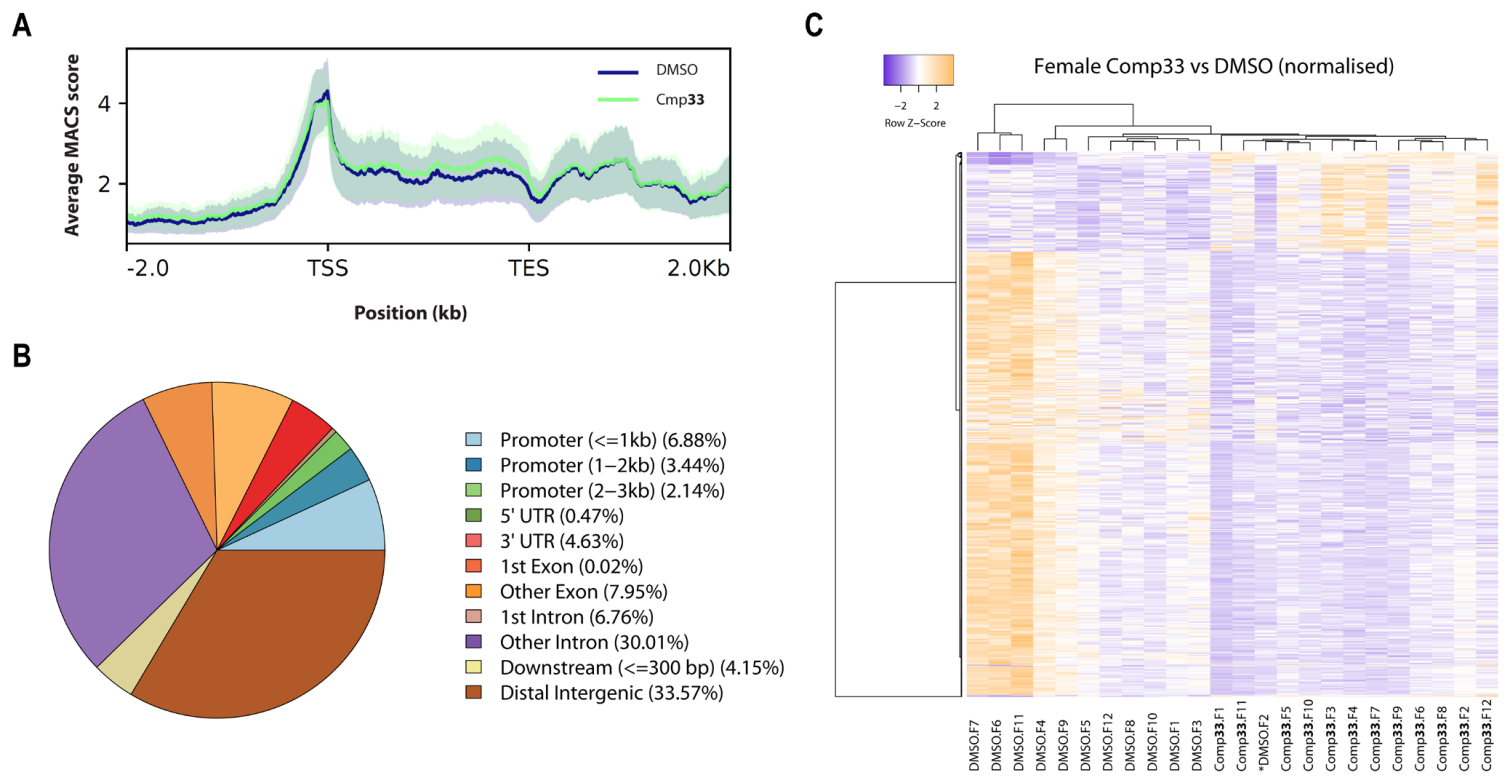
SmLSD1 inhibition affects chromatin structure in adult *S. mansoni* females. ( **A**) - Combined metagene ATAC profiles derived from compound **33** - (green) and mock (DMSO, blue) treated *S. mansoni* adult female worm libraries. The bold lines represent average values (from the 12 replicates), with the standard errors in shades (light green for compound **33**, light blue for DMSO, light grey for the overlapping area of the first two). X-axis in kilobase (kb). TSS = Transcription start site, TES = transcription end site. −2.0 represents 2 kb upstream of the TSS, and 2.0 represents 2 kb downstream of the TES. Y-axis represents the averaged intensity value of the ATAC-Seq enrichment, which is based on the number of aligned reads as well as the average distance between reverse and forward reads that were converted into ‘peak scores’ by MACS and a background correction with Poisson Pvalue (-log10(pvalue) using control as lambda and treatment as observation. ( **B**) - Pie chart illustration of genomic distribution for the ATAC-seq peaks differentially found between mock and compound **33** treated female worms. The percentage of ATAC-seq peaks is provided for each genomic feature: exonic (1 ^st^ exon or other exons), intronic (1 ^st^ intron or other introns), distal intergenic regions (located between genes; greater than 300 bp downstream of gene end and more than 3 kb upstream of its neighbouring gene), promoter region, downstream region (up to 300 bp downstream of gene end). ( **C**) Heatmap of differentially accessible ATAC-seq peaks ( *p*-value < 0.05) in adult female worms derived from DMSO- (n = 12) versus compound **33**-treated female worms (n = 12). Clustering method: average linkage; Distance Measurement Method: Manhattan; clustering applied to rows and columns. Heatmap represents row-based z-scores of DESeq2 normalized Tn5 insertion counts for each differentially accessible ATAC-seq peak. Every line represents a peak, 849 in total. High (ATAC-seq up) and low (ATAC-seq down) chromatin accessibility is indicated in orange and violet, respectively.

To identify a molecular signature underpinning the adult worm phenotypes induced by compound **33**-mediated SmLSD1 inhibition, chromatin structure was assessed in individual male and female worms (n = 12 per treatment) by ATAC-seq ( Figure 9 and *Extended data,* Figure S5 ^38^). The projection of ATAC-seq reads on a metagene profile indicated that Tn5 accessibility was enriched at transcription start sites (TSS), supporting a spatial role for open chromatin regions in transcriptional regulation (similar to previous observations ^65, 99^) ( Figure 9A for females and *Extended data,* Figure S5A ^38^ for males). Moreover, Tn5 accessibility was also observed throughout the gene body (compared to transcriptional end sites, TES) in both compound **33** treated and control worms. The metagene profile also indicated that chromatin accessibility was affected by SmLSD1 inhibition in both sexes ( Figure 9A and *Extended data,* Figure S5A ^38^). Subsequently, ChIPSeeker was used to identify regions where the ATAC-Seq differential signal (between compound **33** and control treated samples) was enriched in both sexes ( Figure 9B and *Extended data,* Figure S5B ^38^). While distal intergenic regions contained a small proportion (33.57% and 24% in female and male, respectively) of ATAC-Seq signals in both sexes, the majority of the differential signals were localised within genes (intragenic regions). Approximately 12.4% and 19.7% of ATAC-Seq signals (in female and male, respectively) were found within the 3 kb region upstream (promoter regions), consistent with the rather euchromatic nature of these regions and thus high Tn5 accessibility ( Figure 9B and *Extended data,* Figure S5B ^38^). However, intronic regions (1 ^st^ intron + other introns) have the highest proportion (about 40%) of ATAC-Seq signal in both males and females.

To further characterise statistically significant changes in chromatin accessibility mediated by compound **33** treatment of adults compared to controls, we analysed the ATAC-Seq data for each male and female individual. Out of 71,859 ATAC-Seq peaks found in females ( *Extended data,* Table S4A ^66^), 843 genomic loci ( *Extended data,* Table S4B ^66^) were found to be significantly affected (1.17%; adjusted *p*-value ≤0.05) by compound **33** treatment. In males, out of 84,997 ATAC-Seq peaks identified ( *Extended data,* Table S4C ^66^), 2,107 loci ( *Extended data,* Table S4D ^66^) were found to be affected (2.48%; adjusted *p*-value ≤0.05). Furthermore, heatmap representation of the ATAC-Seq signal indicated that a strong decrease in chromatin accessibility (violet colour in heatmap in Figure 9C) was observed in compound **33** treated females. Except for one sample (DMSO F2, highlighted by*), control (DMSO) treated, and compound **33** treated female samples were clearly clustered, suggesting the high quality of the sequencing data across a library of 24 samples (each sample was derived from an individual worm). Similar conclusions were drawn from the male dataset ( *Extended data,* Figure S5C ^38^).

Of these statistically enriched ATAC-Seq signals, 87% (732/844) and 76% (1,597/2,107) of the differentially identified loci were found in female and male gene bodies, respectively ( *Extended data,* Table S4E ^66^). Those lists of genes were further segregated depending on whether the ATAC-Seq signals were differentially associated with compound **33** (negative value of log _2_FC - fold change) or control treated samples (positive value of log _2_FC) ( *Extended data,* Table S5A and Table S6A ^66^, female and male respectively), keeping in consideration that unique genes may be associated with multiple ATAC-seq peaks (up to four and 15 different peaks for the female and male datasets, *Extended data,* Table S5B and Table S6B ^66^, female and male respectively). Interestingly, we observed that the majority of the ATAC-seq positive signals (accessible chromatin) were found in the control treated samples (DMSO - 689/844 = 81.6%, *Extended data,* Table S5C for female; 1,549/2,107 = 73.5%, Table S6C for male ^66^) compared to the compound **33** treated samples (155/844 = 18.3%, *Extended data,* Table S5D for female; 557/2,107 = 26.4%, Table S6D for male ^66^). Similar proportions were observed when looking at the number of unique genes ( *Extended data,* Table S5E and S5F for female and Table S6E and S6F for male ^66^). Therefore, these findings indicated that compound **33** treatment (i.e., SmLSD1 inhibition) led to a reduction of DNA accessibility (increase in heterochromatin) in both adult male and female worms.

**Figure 10.**
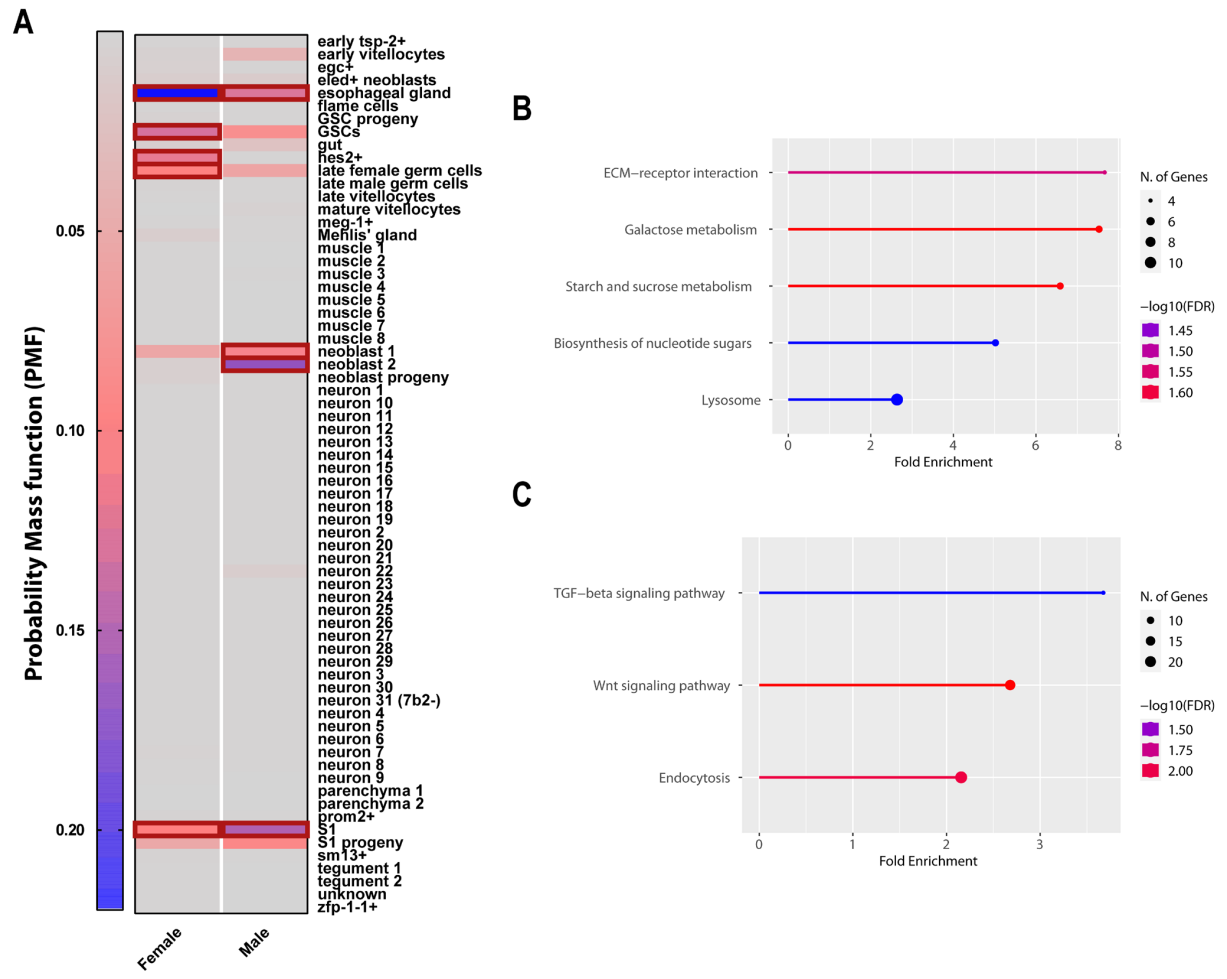
Adult ATAC-seq peaks are enriched in genes differentially expressed in distinct cell clusters and specific KEGG pathways. ( **A**) - A hypergeometric distribution was used to intersect statistically significant ATAC-seq signals (between mock and compound **33** treated worm samples) with genes differentially expressed in 68 adult worm cell clusters. The list of cell clusters is shown on the y-axis. The resulting hypergeometric probability mass function (PMF) scores for female and male genes are represented as heat maps (colour legend indicates significance level, in terms of PMF: grey for PMF<0.05 and blue for PMF>0.2 – cut-off value of PMF>0.1). Cell clusters over-represented (PMF value above 0.1) in female and male samples are highlighted within bold red boxes. ( **B**) KEGG enrichment analyses of the statistically significant ATAC-seq signals within the female samples. ( **C**) KEGG enrichment analyses of the statistically significant ATAC-seq signals within the male samples. The most enriched KEGG signalling pathways are shown here. The most significant processes are highlighted in red, and the less significant processes are highlighted in blue according to -log _10_(FDR) values. Larger dots in the graph indicate a greater number of genes involved. FDR = false discovery rate.

The genes that contained these enriched, intragenic ATAC-Seq signals (732 females and 1,597 male genes - *Extended data,* Table S4E ^66^) were next intersected with the genes found to be differentially expressed (using scRNA-Seq) in the 68 distinct cell clusters identified in adult *S. mansoni* worms by Wendt *et al*. ^100^ ( Figure 10). For both sexes, we performed a hypergeometric distribution analysis on the two datasets (ATAC-seq vs scRNA-Seq) to calculate the probability that the ATAC-Seq signal is over-represented in genes differentially expressed within distinct cell clusters. In females, ATAC-Seq loci affected by compound **33** treatment were over-represented (PMF > 0.1, outlined in bold boxes) in genes differentially expressed/enriched in the esophageal glands, the germinal stem cells (within the ovary), the hes2+ stem cells (a neoblast progenitor population), the S1 stem cells (within the vitellaria) and the late female germinal stem cells within the ovary ( Figure 10A). The genes *smp_312640* (expression enriched in the late female germ cell cluster) and *smp_200410* (expression enriched in both GSC clusters) represent two examples where compound **33** statistically affects ATAC-Seq signals found in the intragenic region (3´- UTR and intron, respectively as shown in *Extended data,* Figure S6 ^38^). In males, ATAC-Seq loci were found to be over-represented in genes differentially expressed in neoblasts (neoblast 1 and 2 populations), the esophageal gland and a small population of S1 cells previously described in this sex ( Figure 10A) ^96, 101^.

After having established that there were significant differences in chromatin accessibility between compound **33** treated and control treated individuals, we asked whether these differences might also affect specific gene functions or pathways. Consequently, KEGG analysis of the ATAC-Seq signal was performed for both sexes. In females, the ATAC-seq signal was most significantly enriched in genes associated with sugar metabolism, lysosomal biology and extracellular matrix (ECM)-receptor interactions ( Figure 10B). In males, the ATAC-seq signal was associated with genes involved in transforming growth factor (TGF)-beta and Wnt signalling pathways as well as endocytosis ( Figure 10C). While these results do require further validation, it is tempting to speculate that at least some of the adult worm phenotypes observed upon compound **33** treatment are influenced by the dysregulation of chromatin structure and coordinated gene expression associated with these KEGG functional categories.

### Compound 33 likely inhibits SmLSD1 activity by covalently reacting with the FAD cofactor

**Figure 11.**
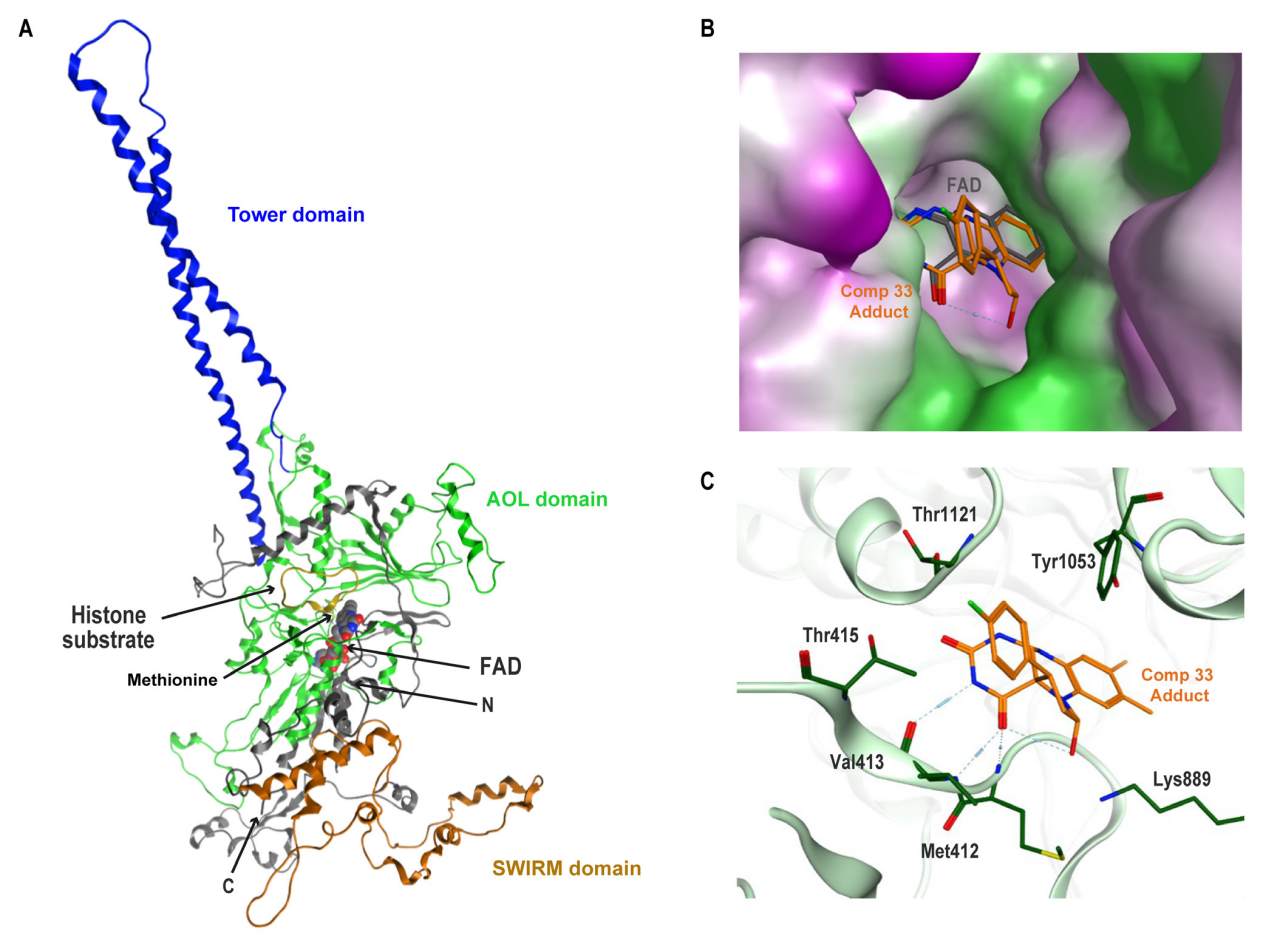
Proposed binding mode of the compound 33-derived adduct in SmLSD1’s active site. ( **A**) - Ribbon diagram representation of full-length Smp_150560’s (SmLSD1) homology model: unstructured N-terminal region (N) in grey, SWIRM domain in orange, AOL domain in green and Tower domain in blue. The C-terminus is indicated (C); the cofactor FAD is shown as spheres (grey for carbons, red for oxygen, blue for nitrogen); the histone protein is represented as a yellow ribbon; the methionine is shown as a yellow stick. ( **B**) - Surface diagram of SmLSD1 showing the FAD−PCPA adduct of compound **33** in the protein’s active site. The binding mode of this adduct is compared to the orientation of the cofactor FAD (here the structure of the cofactor was reduced at only the flavin ring). The adduct and the flavin ring of FAD are shown as a stick model in orange and dark grey, respectively. The protein surface was coloured by lipophilicity with purple, white and green representing hydrophilic, neutral and lipophilic regions, respectively. ( **C**) - Ligand interactions of the compound **33** covalent adduct with highlighted conserved active site residues of SmLSD1. Amino acid lateral chains involved in interactions are shown as dark green sticks and labelled according to their position in the full-length amino acid sequence of Smp_150560 (shown as light green ribbon).

Irreversible inhibitors of LSD1 (tranylcypromine and its derivatives) have been shown to modify the FAD cofactor by covalent bonding of their cyclopropylamine group to the N5 atom of the cofactor flavin ring. Hence, the resulting N5 adduct inhibits the demethylase activity of the enzyme ^24^. Due to the structural similarity of compound **33** with these tranylcypromine derivatives ^102– 104^, a similar molecular mechanism of action was expected upon interaction of this compound with SmLSD1. To explore this, homology modelling and compound docking were performed ( Figure 11).

 As previously published ^19^, the homology model of the parasite enzyme was generated using the elucidated HsLSD1 structure (PDB entry: 2V1D, Figure 11A) ^105^. The predicted 3D model of SmLSD1 shows the main features of this protein, particularly the catalytic domain (Amino Oxidase-Like domain - AOL domain shown in green) containing the histone substrate (in brown), cofactor binding pocket and the Tower domain (in blue) with its antiparallel coiled-coil conformation ^106^.

In order to predict the binding mode of compound **33** ( *Extended data,* Figure S7A ^38^) to SmLSD1, the N5 adduct of this compound with the flavin ring of the FAD ( *Extended data,* Figure S7B ^38^) cofactor was prepared ( *Extended data,* Figure S7C ^38^) and used as a ligand for molecular docking to the SmLSD1 homology model. The predicted docking pose fitted very well in the active site of SmLSD1 with the flavin ring of the adduct superimposed on the same tricyclic ring of the FAD cofactor ( Figure 11B). A closer analysis of the ligand-protein interactions ( Figure 11C) revealed many contacts between the flavin ring of the chemical adduct and amino acid residues, which are typically involved in the cofactor binding to the N-terminal region of the amino-oxidase like domain in LSD1 (Met412, Val413 and Thr415). The fluoro-phenyl ring of the FAD-adduct extended outside the active site, towards the substrate binding pocket and was embedded between two residues (Tyr1053 and Thr1121, Figure 11C). These two residues together define the aromatic cage, which has been described for other amine oxidase enzymes (including LSD1) and contributes to active site hydrophobic shielding from the influx of external solvents ^107, 108^. In addition to this, we also observed the orientation of the compound towards another conserved residue, the invariant lysine (Lys889), which has been investigated for its role in catalysis as well as in proton transfer (acting as an active-site base of LSD1) ^109, 110^.

## Discussion

Studies of the *S. mansoni* lifecycle have shown that transitions between, and development within, both intermediate and definitive hosts are finely regulated by epigenetic factors ^17, 18, 23, 111– 113^. While a critical role for histone methylation and demethylation in these processes has been demonstrated ^19– 21, 23^, the specific contributions of enzymes catalysing these reversible post-translational modifications have not been thoroughly characterised. Following on from our previous investigations demonstrating that anti-neoplastic anthracyclines could bind to the target pocket of SmLSD1 and kill multiple schistosome developmental stages ^19^, we decided to evaluate the anti-schistosomal properties of 39 HsLSD1 inhibitors.

Initial *ex vivo* schistosomula screening of the 39 compounds identified five (compounds **15**, **16**, **33**, **35** and **36**) with activity (EC _50_ below 10 µM; Figure 2) similar to that previously found for the putative SmLSD1 inhibitors daunorubicin hydrochloride (EC _50_ below 6 µM) and pirarubicin (EC _50_ below 3 µM) ^19^. When the *ex vivo* screens were expanded to adult parasites ( Figure 3), the most active anti-schistosomula compound (compound **33**) also demonstrated the strongest effects (decreased worm motility and reduction in IVLE production; Figure 3). While a reduction in adult worm viability and IVLE production was also observed for a recently described and structurally-related LSD1 inhibitor MC3935 ^21^, the activity of compound **33** reported here was more potent.

To understand potency differences of the 39 tested compounds, physiochemical properties such as molecular weight (MW) and lipophilicity (in terms of calculated LogP) were compared ( *Extended data,* Figure S8 ^38^). Most of the compounds have a molecular weight between 400 and 600 g/mol, but variable lipophilicities. Amongst the tested compounds, those with the greatest anti-schistosomal activities (compounds **15**, **16**, **33**, **35** and **36**) also were some of the most lipophilic ones. It is tempting to speculate that increased lipophilicity facilitates greater penetrance of anti-schistosomal compounds; this physiochemical property may be an important anthelmintic feature when considering the parasite’s heptalaminate surface membranes ^114^.

The presence of a para-fluorophenyl substitution on the cyclopropyl ring of the tranylcypromine derivatives was identified as a common feature of the five most active compounds ( *Extended data,* Table S7 ^66^). Considering the mechanism of action of these compounds, they are all capable of forming a covalent adduct with FAD that contains the fluorophenyl ring. Though the para-fluoro substitution may be important for potency, this structural feature cannot explain the differences in activity among the five most active compounds. These conclusions align to the current literature on N-alkylated 2-PCPA analogues (i.e., analogues with substituents on the amine group of the tranylcypromine scaffold), which demonstrate enhanced potency and selectivity towards LSD1 compared to other PCPA derivatives. Nevertheless, the mechanism (s) by which N-alkylated 2-PCPA derivatives inhibit LSD1 is still not well understood as the N-substituents do not contribute to the adduct formed with FAD ^115, 116^. However, exploring structure-activity relationships (SAR) within our study, two compound subfamilies were identified; the first subfamily (including compounds **15**, **16** and **33**) contains an alkyl linker between the cyclopropyl ring and a pyrazine ring, which is replaced by a pyrimidine ring in the second subfamily (including compounds **35** and **36**). The *in silico* molecular docking of these five compounds to SmLSD1’s active site highlighted a more favourable docking score (XP score, *Extended data,* Table S7 ^66^) for compound **33;** this is likely due to the presence of a benzyl group conferring more flexibility when compared to the other compounds. This structural feature (combined with increased lipophilicity) could explain the greater anti-schistosomal activity of compound **33** compared to other SmLSD1 inhibitors assessed in this study (e.g., *Extended data,* Table S7 ^66^).

Reassuringly, compound **33**-induced inhibition of IVLE production broadly recapitulated the previously described RNAi-mediated knock down phenotypes of *smlsd1* ( *smp_150560*) in adult worms and the viability assays of other synthetic LSD1 inhibitors ^19, 21, 53^. In addition to an egg laying deficiency, compound **33** treatment resulted in the presence of significantly less Fast blue BB ^+^ vitelline droplets (compared to controls) and, by inference, affected the ability of adult females to generate mature vitellocytes (vitelline droplet-containing S4 cells) and to package them into eggs ( Figure 6, Figure 7 and *Extended data,* Figure S3 ^38^). These collective phenotypes pointed to a global defect in the female’s reproductive system (ootype, vitellaria and ovary), which was further supported by compound **33**’s complete inhibition of gonadal and vitellaria stem cell (S1) proliferation ( Figure 8). The additional inhibition of gonadal stem cell proliferation in the testes ( *Extended data*, Figure S4 ^38^) following compound treatment suggests an essential contribution of SmLSD1 in both female and male germline tissues (leading to defects in oogenesis, vitellogenesis and spermatogenesis). In support of this contention, it was previously demonstrated that mutants of *spr 5* ( *lsd1* homologue) in *Caenorhabditis elegans* led to progressive sterility in progeny due to dis-regulation of spermatogonia-associated genes ^117^. When taken together, our data validate previous observations ^16^ and highlights an essential role for epigenetic regulation in oviposition and *Schistosoma* reproductive biology at large.

Similar to its effect on germinal stem cells, compound **33** also inhibited adult neoblast proliferation in both sexes ( Figure 8 and *Extended data,* Figure S4 ^38^). These proliferation defects are comparable to those observed in mammalian *lsd1*-expressing neural stem cells treated with the LSD1 inhibitors pargyline or tranylcypromine ^98^ and support previous findings confirming that *Smlsd1* is also expressed in rapidly dividing cells throughout the parasite ^64^. *Smmbd2/3* (encoding an epigenetic reader of 5-methylcytosine) and *Smcbx* (encoding an epigenetic reader of methyl lysine) are also co-expressed in proliferative schistosome cells ( *h2b+*); similar to SmLSD1 inhibition, knockdown of either reader results in reduced neoblast proliferation ^118^. When considered alongside neoblast proliferation defects found in adult schistosomes treated with 5-azacytidine (a DNA methyltransferase inhibitor ^15, 60^), epigenetic processes are rapidly emerging as essential regulators of schistosome stem cell biology.

In addition to affecting schistosomula and adult worm phenotypes, we also demonstrated that compound **33** markedly reduced the transformation of miracidia into germinal cell-enriched sporocysts ( Figure 5) ^119^. A ‘block in transformation’ phenotype was also observed when the histone methyltransferase inhibitors A366 and GSK343 (likely targeting G9a/GLP and EZH1/H2 homologs respectively ^23^) were used as part of studies investigating the role of H3K27 methylation during schistosome lifecycle progression ^23^. These results, derived from distinct studies of different histone methylation and demethylation components (G9a/GLP and EZH1/H2 HMTs ^23^ vs LSD1 HDM here), mutually support a critical role for histone methylation regulation (on both H3K4 and H3K27) in miracidium to sporocyst transformation. When further considering the activity of compound **33** on juvenile worms ( Figure 4), collectively, our data suggests that targeting SmLSD1 with small molecules represents a strategy capable of affecting many (if not all) schistosome lifecycle stages. LSD1 inhibitors currently in clinical development for the treatment of hematologic malignancies and solid tumours have been shown to be relatively safe and well tolerated ^83^, suggesting that LSD1 inhibitors (or derived compounds) may be similarly tolerated in people affected by schistosomiasis.

Further evidence (in support of *in silico* docking, Figure 11) that compound **33** inhibits SmLSD1 function is derived from ATAC-seq analyses of adult schistosomes. Firstly, most of the differential ATAC-Seq signal observed between compound **33** treated and control adults (regardless of sex) was found in the intragenic regions, with a higher proportion in the introns (first and other introns - Figure 9 and *Extended data,* Figure S5 ^38^). Enrichment of ATAC-Seq signals to similar genomic features was recently found in murine germinal centre (GC) B cells deleted for *lsd1* ^120^. This suggests that Tn5 chromatin accessibility to introns and intergenic regions in SmLSD1 inhibited adult schistosomes is equivalent to *lsd1* depleted murine GC B cells.

Secondly, compound **33** is structurally very similar to another potent human LSD1 inhibitor (MC3935), which was previously shown to also be active against *S. mansoni* schistosomula and adult worms ^21^. MC3935 is a synthetic 2-PCPA derivative, which blocks the enzymatic activity of LSD1 by formation of a covalent drug-protein adduct as confirmed by crystallographic studies with closely related structural analogues (MC2580 and MC2584, PDB 2XAS ^121^ and 2XAQ ^122^, respectively). MC3935-mediated inhibition of SmLSD1 demethylase activity was confirmed by western blot analysis of soluble protein extracts probed with antibodies specific for H3K4me1 or H3K4me2 marks (LSD1-specific histone marks). MC3935 treatment led to an accumulation of these LSD1 marks when compared to the DMSO controls. As MC3935 and compound **33** are structurally related (and likely to operate through a similar mechanism of action), we contend that compound **33** exerts a similar effect on H3K4me1 or H3K4me2 marks. While our efforts to quantify this experimentally were affected by H3 clipping ^123, 124^, H3K4me2 marks appeared to be more abundant in compound **33** treated male worms compared to controls ( *Extended data,* Figure S9 ^38^).

The ATAC-Seq data also suggest a molecular mechanism underpinning how SmLSD1 inhibition affects adult schistosome oviposition ( Figure 3). Specifically, the genomic loci that have altered accessibility upon compound **33** treatment (i.e. SmLSD1 inhibition) in adults are statistically over-represented (by hypergeometric analyses) in genes that are differentially expressed in female germinal stem cells, hes2+ neoblast progeny, S1 stem cells of the vitellaria and late germinal stem cells of the ovary as well as male neoblast 1/2 stem cells and S1 stem cells ^100, 101^ ( Figure 10). Thus, compound **33** induced alterations to the chromatin in these loci likely affect key functions underpinning schistosome cell proliferation and differentiation leading to oviposition defects. The complementary phenotypes observed in compound **33** treated adults supports this contention ( Figure 6, Figure 7, Figure 8, *Extended data,* Figure S3 and Figure S4 ^38^). Interestingly, in both sexes, SmLSD1 inhibition also affects chromatin structure of genes differentially expressed in the esophageal gland ( Figure 10). This finding could highlight a role for SmLSD1 in controlling the alternative splicing/protein variation of micro exon genes ^125^, which are found differentially expressed in large numbers in this gland (supporting material of Wendt *et al.* ^100^). Validation of this contention would require further investigation and is beyond the scope of this study.

KEGG enrichment analyses identified that the differential ATAC-seq signal in females was most significantly enriched in genes associated with sugar metabolism, lysosomal biology and ECM-receptor interactions ( Figure 10B). As these pathways contribute to nutrient acquisition/synthesis/storage and energy production ( 126, 127), it is tempting to speculate that chromatin structure alterations leading to dysregulated gene function in these enriched KEGG categories likely is responsible for many of the compound **33** mediated female phenotypes observed in our study. In males, enrichment of differential ATAC-seq signal in genes linked to transforming growth factor (TGF)-beta and Wnt signalling pathways as well as endocytosis was observed ( Figure 10C). While the schistosome TGF-β signalling pathway has been implicated in parasite development, host interactions, maturation of vitelline cells and embryogenesis of the egg ^128, 129^, the Wnt pathway has not been as extensively characterised ^130– 132^. A few detailed investigations show that G-quadruplex (G4) structures are enriched in genes encoding Wnt pathway components ^133^ and that schistosome orthologous to planarian *wnt2* are expressed in a spatial gradient along the worm posterior suggesting a linkage to axial development ^96^. In a recent scRNA-seq study of schistosomula, a muscle cluster with a putative positional function contained over-expression of *wnt-2* (Smp_167140) and further supports a role for the Wnt pathway in schistosome development ^134^. Other reports exploring Wnt signalling in schistosomes are focused on *S. japonicum* Wnt4 ^135^ and Wnt5 ^106^. *Sjwnt5* is found highly expressed in testes, ovaries and vitellaria suggesting the involvement of Wnt signalling in the development of the reproductive organs of both sexes. Understanding how histone methylation and G4s affect the Wnt pathway and schistosome development represent an attractive area of future investigation.

The protein-coding DNA content of the *S. mansoni* genome is ~ 5% ^136^ in comparison to the protein-coding fraction of prokaryotic (80–90%) and mammalian genomes (1.22%) ^137, 138^. Despite representing a small portion of the entire parasite genome, approximately three quarters of the ATAC-seq signals occurred within or in close proximity to genes when compared to the signals found in intergenic regions (33.57% and 24% in female and male, respectively). From our analysis of chromatin accessibility, it is clear that SmLSD1 inhibition leads to an accumulation of heterochromatin (i.e., less accessible DNA). This chromatin perturbation likely impacts gene expression regulation and drives the phenotypes observed in the adults (as well as other lifecycle stages) upon SmLSD1 inhibition.

The present work, progressing investigations of *S. mansoni* epigenetics, focuses on the biological and epigenomic implications induced by chemical modulation of SmLSD1 (Smp_150560). Our findings provide a rationale for repurposing compounds originally developed against human targets for use as anti-schistosomals ^139^. This work, alongside others ^19, 21^, validates SmLSD1 as a putative target for schistosomiasis treatment.

Since its first application in 2013 ^140^, ATAC-seq has gained greater acceptance as an approach to dissect epigenetic landscapes ^141– 143^. To date, this is one of the first applications of ATAC-Seq in a non-model species ^144^ and represents the very first one to show its suitability in characterising the effect of epigenetic modulators during drug discovery investigations of schistosomiasis. Further use of this and other genome biology tools (e.g., ChIP-Seq ^145, 146^) to explore chromatin physiology in schistosomes treated with epi-drugs should advance our molecular understanding of the fundamental biology of this neglected tropical disease-causing pathogen.

## Data Availability

Bioproject: ATAC-seq on drug treated Schistosoma mansoni adult worms. Accession number PRJNA830864, https://identifiers.org/bioproject:PRJNA830864 ^147^. This project contains the raw Fastq data. ATAC-seq data can be visualised via Integrative Genomics Viewer (IGV) web browser here. The search function requires the locus position and this is defined as the following string: ‘Chrom’:’Start’-‘End’ (for example ‘SM_V7_2:9296729-9297279’) where ‘Chrom’ represents the chromosome position, ‘Start’ and ‘End’ represent start and end position of the ATAC-seq peak, respectively. This information can be found in the *Extended data* Tables S5C/S5D/S6C/S6D ^66^. Figshare: Chemical modulation of *Schistosoma mansoni* lysine specific demethylase 1 (SmLSD1) induces wide-scale biological and epigenomic changes – Supplementary Figures. https://doi.org/10.6084/m9.figshare.21814710 ^38^. This project contains the following extended data: Figure S1.pdf -Dose response titrations of compounds 15, 16, 33, 35 and 36 against schistosomula. Figure S2.pdf - Compound 33 treatment induces the release of oocytes, spermatozoa and vitelline cells from adult worms. Figure S3.pdf - Fast Blue BB stain (orange-red labelling) showing loss of mature vitellocytes in compound 33-treated female worms compared to the control ones. Figure S4.pdf - Compound 33 treatment reduces stem cell proliferation in adult male worms.) FigureS5.pdf - SmLSD1 inhibition causes changes in chromatin structure in S. mansoni male adult worms. Figure S6.pdf - Visualisation of ATAC-seq samples. Figure S7.tif - Computational preparation of the covalent adduct derived from the interaction of compound 33 with the FAD cofactor. Figure S8.pdf - Chemical space covered by the library of 39 HsLSD1 inhibitors. Figure S9.pdf - Compound 33 treatment inhibits H3K4me2 demethylation in adult male worms. The project also contains raw microscope images related to: Figure 6, 7 and 8 Figure S2, S3, S4 and S9 Figshare: Chemical modulation of *Schistosoma mansoni* lysine specific demethylase 1 (SmLSD1) induces wide-scale biological and epigenomic changes - Supplementary tables and movies. https://doi.org/10.6084/m9.figshare.21814623 ^66^. This project contains the following extended data: Table S1.xlsx - Chemical structures of the 39 compounds included in this study. Table S2.xlsx - Z´ values for both phenotype and motility of the Roboworm screens performed on the 39 compounds. Table S3.xlsx - Z´ values for both phenotype and motility of the Roboworm screens performed on the titration of the five selected compounds (compounds **15**, **16**, **33**, **35** and **36**). Table S4A.xlsx - List of the 71,859 ATAC-Seq peaks found in the female dataset Table S4B.xlsx - List of corresponding 844 genomic loci associated to statistically significant ATAC-seq differences (adjusted *p*-value ≤0.05) following compound **33** treatment (female dataset). Table S4C.xlsx - List of the 84,997 ATAC-Seq peaks found in male dataset. Table S4D.xlsx - List of corresponding 2,107 genomic loci associated to statistically significant ATAC-seq differences (adjusted *p*-value ≤0.05) following compound **33** treatment (male dataset). Table S4E.xlsx - List of female and male genes associated to statistically significant ATAC-seq differences between compound **33** and mock treatments. Table S5A.xlsx - List of the 844 genomic loci associated to statistically significant ATAC-seq differences (female subset). The table contains the gene ID, chromosome position, start and end of the ATAC-seq peak position, ATAC-seq peak identifier, value of log2-foldchange (log2FC). Table S5B.xlsx - Occurrence of ATAC-seq peaks (accessible chromatin) per genes (female subset). The table contains the gene ID and the associated number of ATAC-seq peaks. Table S5C.xlsx - List of ATAC-seq positive signals (accessible chromatin) found in the mock female samples (filtered from S5A Table with log _2_FC>0). Table S5D.xlsx -List of ATAC-seq positive signals (accessible chromatin) found in the compound **33**-treated female samples (filtered from S5A Table with log _2_FC<0). Table S5E.xlsx -List of female genes containing higher ATAC-seq positive signals (accessible chromatin) in compound **33**-treated samples. The table contains the gene ID, the number of associated ATAC-seq peaks and the average log _2_FC across all the peaks associated with each gene (log _2_FC<0 corresponds to higher ATAC-seq positive signals found in the compound **33** treated samples). Table S5F.xlsx -List of female genes containing higher ATAC-seq positive signals (accessible chromatin) in mock samples. The table contains the gene ID, the number of associated ATAC-seq peaks and the average log _2_FC value across all the peaks associated to each gene (log _2_FC>0 corresponds to higher ATAC-seq positive signals found in the control treatment). Table S6A.xlsx -List of corresponding 2,107 genomic loci associated with statistically significant ATAC-seq differences (male subset). The table contains the gene ID, chromosome position, start and end of the ATAC-seq peak position, ATAC-seq peak identifier, value of log _2_-foldchange (log2FC). Table S6B.xlsx -Occurrence of ATAC-seq peaks per genes (male subset). The table contains the gene ID and the associated number of ATAC-seq peaks. Table S6C.xlsx -List of ATAC-seq positive signals (accessible chromatin) found in the mock male samples (filtered from S6A Table with log _2_FC>0). Table S6D.xlsx -List of ATAC-seq positive signals (accessible chromatin) found in the compound **33**-treated male samples (filtered from S6A Table with log _2_FC<0). Table S6E.xlsx -List of male genes associated with higher ATAC-seq positive signals (accessible chromatin) in compound **33**-treated samples. The table contains the gene ID, the number of associated ATAC-seq peaks and the average log _2_FC across all the peaks associated to each gene (log _2_FC<0 corresponds to higher ATAC-seq positive signals found in the compound **33** treated samples). Table S6F.xlsx -List of male genes associated with higher ATAC-seq positive signals (accessible chromatin) in mock samples. The table contains the gene ID, the number of associated ATAC-seq peaks and the average log _2_FC value across all the peaks associated with each gene (log _2_FC>0 corresponds to higher ATAC-seq positive signals found in the control treatment). Table S7.xlsx -List of the structure, the docking score and EC _50_ values (on schistosomula and adult worms) of the five most active compounds. Table S8.xlsx -List of 24 adult worm ATAC-seq sample IDs, primer sequences and additional PCR cycles used for preparation of ATAC-seq libraries. Table S9.xlsx -Excel workbook used for the hypergeometric test for overrepresentation of ATAC-Seq signal within the available scRNA-Seq data derived from adult worms ^100^. Movie S1.mp4 -Video of *S. mansoni* compound **33**-treated (3.13 µM, right-hand side) and control (DMSO, left-hand side) worm pairs after 72 h incubation in tissue culture wells. Notice the lack of parasite attachment and the presence of cellular material within the compound treated well. Movie S2.mp4-Serial optical sections of DAPI-stained, *S. mansoni* egg. Comparison between the compound **33**-treated (3.13 µM, right-hand side) and the negative control (DMSO, left-hand side) egg is provided. Movie S3.avi-Serial optical sections of *S. mansoni* adult female worm stained with DAPI and EdU. In these series of optical sections, three different anatomical regions (anterior region, gonadal system and posterior region, from left to right) of the worm were observed. Comparison between the negative control (DMSO, first row) and the compound **33**-treated (3.13 µM, second row) parasites is provided. Movie S4.avi-Serial optical sections of *S. mansoni* adult male worm stained with DAPI and EdU. In these series of optical sections, three different anatomical regions (anterior region, gonadal system and posterior region, from left to right) of the worm were observed. Comparison between the negative control (DMSO, first row) and the compound **33**-treated (3.13 µM, second row) parasites is provided. Figshare: ARRIVE checklist for ‘Chemical modulation of Schistosoma mansoni lysine specific demethylase 1 (SmLSD1) induces wide-scale biological and epigenomic changes.’ https://doi.org/10.6084/m9.figshare.21814710 ^38^. Data are available under the terms of the Creative Commons Attribution 4.0 International license (CC-BY 4.0).

## Software availability

Archived source code at time of publication: https://doi.org/10.5281/zenodo.3929417 ^51^.

License: GNU General Public License version 2 (GPL-2.0)
