# Supplementary material for: Chemical modulation of Schistosoma mansoni lysine specific demethylase 1 (SmLSD1) induces wide-scale biological and epigenomic changes
Source: Wellcome Open Res. 2023 Mar 30;8:146. [Version 1] doi: 10.12688/wellcomeopenres.18826.1 (PMC10375057; doi:10.12688/wellcomeopenres.18826.1)
Supplement: Peer review history

# Peer review: Reviewer response for version 1

**DOI:** 10.21956/wellcomeopenres.20872.r62556

**Published:** 2023-08-17

**Authors:** Klaus Brehm

**Affiliations:** 1 Consultant Laboratory for Echinococcosis, Institute of Hygiene and Microbiology, University of Würzburg, Würzburg, Germany

Padalino *et al.* report work carried out on epigenetic histone modification in the human parasite Schistosoma mansoni. Schistosoma sp. are the cause of devastating disease in humans, which are listed among the Neglected Tropical Diseases, and against which praziquantel is currently the only choice for effective treatment. Due to the threat of widespread resistance of schistosomes towards praziquantel, the opening of new avenues of target mechanisms for the development of parasiticidal anti-infectives is highly important and, in the present work, the authors logically suggest epigenetic control mechanisms that govern parasite stem cell biology and developmental processes as one of these promising mechanisms.

 The authors initially carried out a screen of a library of 39 approved or experimental compounds that have originally been developed to inhibit the human enzyme LSD1, which is a demethylase that removes important modifications from histones. 5 of these compounds proved to be active at a concentration of 3 - 10 μM against schistosomules and, in further assays, significantly affected adult worm survival, motility, and egg production (the actual problem in schistosomiasis). The most effective inhibitor, called compound 33, was further demonstrated to significantly affect juvenile worms, miracidia transformation, and the female reproductive system at low concentrations. From these overall screens against different developmental stages, the authors went to unravel the cellular basis of the effects of compound 33 and demonstrated that it significantly impairs parasite stem cell proliferation. Clear indications for a modulation of epigenetic histone modification by compound 33 were then obtained through ATAC-Seq approaches showing that the compound disrupts chromatin structure in adult worms and particularly affects genes involved in developmental transition processes (encoding, amongst others, wnt-, and TGFβ-signalling components), relevant for stem cell function and differentiation. Concerning the mechanistic basis for these effects, the authors also showed that compound 33 likely inhibits the schistosome ortholog, SmLSD1, by reacting with the important FAD cofactor. Overall, the results obtained by the authors are highly interesting and relevant for studying the role of epigenetic histone modifications in schistosomes. Although compound 33 might not (yet) be a feasible alternative to praziquantel for parasite treatment, it will surely serve as a highly useful tool for molecular and cell biological studies into schistosome epigenetics.   

 The experiments have been carried out in a highly professional manner by researchers with tremendous experience in the system, are described in great detail, and led to convincing data. The results are nicely presented, including easily comprehensible tables, figures, and videos, and are competently discussed. Likewise, the manuscript is very well written, and the work will surely be appealing to researchers working on epigenetic control mechanisms, not only in parasitic flatworms but also beyond (see e. g. the elegant ATAC-Seq approach).

 It would have been nice had the authors discussed a bit more detailed how (whether) compound 33 could also be used to approach epigenetic modifications in other flatworm parasite systems (e.g. Fasciola and cestodes). Overall, however, I want to congratulate the authors on a well performed, interesting, and fine piece of work.

Is the work clearly and accurately presented and does it cite the current literature?

Yes

If applicable, is the statistical analysis and its interpretation appropriate?

Yes

Are all the source data underlying the results available to ensure full reproducibility?

Yes

Is the study design appropriate and is the work technically sound?

Yes

Are the conclusions drawn adequately supported by the results?

Yes

Are sufficient details of methods and analysis provided to allow replication by others?

Yes

Reviewer Expertise:

flatworm parasite molecular and cell biology; developmental biology; genomics

I confirm that I have read this submission and believe that I have an appropriate level of expertise to confirm that it is of an acceptable scientific standard.

# Peer review: Reviewer response for version 1

**DOI:** 10.21956/wellcomeopenres.20872.r62557

**Published:** 2023-07-27

**Authors:** Giovina Ruberti

**Affiliations:** 1 Institute of Biochemistry and Cell Biology, Italian National Research Council (IBBC-CNR), Rome, Italy

The authors assessed the anti-schistosomal activity of 39 Homo sapiens Lysine Specific Demethylase 1 (HsLSD1) inhibitors on different parasite life cycle stages. By *in* *vitro* whole organism assays, compound **33** was identified as the most potent hit impacting: viabilities of schistosomula, juvenile, miracidia and adult parasites; oviposition, ovary and vitellarian architecture and gonadal/neoblast stem cell proliferation.

 By homology modelling and compound docking, they were able to demonstrate that compound **33 **acts like other inhibitors of lysine specific demethylases by covalently forming an adduct with FAD cofactor.

 Moreover, the authors investigated the mechanisms of action of the compound and convincingly show that compound **33** modulates chromatin structure. Molecular and bioinformatics analysis highlighted that chromatin modulation involves genes differentially expressed in specific cell populations. The authors report in adult parasites a statistically over-representation of genes that are differentially expressed in female germinal stem cells, hes2+ neoblast progeny, S1 stem cells of the vitellaria and late germinal stem cells of the ovary as well as male neoblast 1/2 stem cells and S1 stem cells. The molecular and bioinformatics findings can explain some of phenotypical alterations observed in the parasites after compound treatment.

 This is a very interesting paper that highlights the relevance of epigenetics in *S. mansoni *development and femal reproductive system functions. Modulation of SmLSD1 with compound **33 ** validated SmLSD1 as a target for schistosomiasis treatment. Repurposing compounds originally developed against human targets could be considered.

 I really appreciated the detailed description of methods and results and the multidisciplinary approaches used for the study of the LSD1 compounds. The quantitative analysis of the phenotypical modifications in the different stages of *S. mansoni* development after treatment and the molecular studies strongly support the conclusions. The supplementary ATAC-Seq data and the video of the manuscript are also relevant components of this article.

Is the work clearly and accurately presented and does it cite the current literature?

Yes

If applicable, is the statistical analysis and its interpretation appropriate?

Yes

Are all the source data underlying the results available to ensure full reproducibility?

Yes

Is the study design appropriate and is the work technically sound?

Yes

Are the conclusions drawn adequately supported by the results?

Yes

Are sufficient details of methods and analysis provided to allow replication by others?

Yes

Reviewer Expertise:

Schistosome biology and drug discovery using phenotypical and biochemical approaches

I confirm that I have read this submission and believe that I have an appropriate level of expertise to confirm that it is of an acceptable scientific standard.

# Peer review: Reviewer response for version 1

**DOI:** 10.21956/wellcomeopenres.20872.r55971

**Published:** 2023-04-06

**Authors:** Philip T LoVerde

**Affiliations:** 1 Department of Biochemistry and Structural Biology, University of Texas Health Science Center at San Antonio, San Antonio, TX, USA

Schistosomiasis is a neglected tropical disease of global health importance. There is currently only one method of treatment, the drug Praziquantel (PZQ). Due to the danger of resistance to the monotherapy Praziquantel, developing a novel drug with a different mode of action will have a significant impact on global human health and lead to improved treatments for *Schistosoma, *especially in sub-Saharan Africa where 90% of the over 200 million cases occur *.*

 This manuscript focuses on the *S. mansoni* epigenetics, specifically on repurposing compounds originally developed against human targets for use as an anti-schistosomal compound. The target is *S. mansoni* Lysine Specific Demethylase 1 (SmLSD1). The authors performed a screen with a library of 39 HsLSD1 inhibitors against various schistosome life stages and identified one compound, compound 33, that was highly effective against schistosomula, immature intramammalian stages, adult worms and miracidia. Compound 33 blocked the transition of miracidia to sporocyst, an intramolluscan stage. As a research tool this would be of interest but not as an antischistosomal drug. However, the demonstration that Compound 33 interferes with the male-female interaction that impacts vitelline cell development, ovarian architecture, gonadal stem cell proliferation all interfere with female reproductive activity and thus block egg production which is responsible for the pathogenesis and transmission of schistosomiasis is very significant.

 The experiments to produce the supporting data were all extremely well-designed, reproducible, controlled and analyzed. The experiments are presented in such detail that they can be reproduced with other target multicellular eucaryotic parasites. The data generated by this study are available in detail in supplementary figures, tables and movies. The authors also focused on the mechanism of action. Using homology modelling and compound docking, they were able to produce convincing evidence that compound 33 acts like other inhibitors of lysine specific demethylases by covalently forming an adduct with FAD cofactor.

 Finally, they adapted ATAC-seq as an approach to characterize statistically significant changes in chromatin accessibility mediated by compound **33** treatment of adult male and female schistosomes compared to controls. They were able to demonstrate that that compound **33** treatment led to a reduction of DNA accessibility (increase in heterochromatin) in both adult male and female worms. Analysis of the results led to the conclusion that these alterations resulted in a global defect in the female’s reproductive system (ootype, vitellaria and ovary) and thus egg production. This result was further supported by compound **33**’s complete inhibition of gonadal and vitellaria stem cell (S1) proliferation. Evidence also demonstrated that inhibition of gonadal stem cell proliferation in the testes indicated an essential contribution of SmLSD1 in both female and male germline tissues. 

 Overall, the outcomes of the research meticulously described in this paper significantly increase our understanding of female reproductive biology and schistosome biology in general. The data clearly demonstrate that *Schistosoma mansoni* lysine specific demethylase 1 (SmLSD1) is a viable drug target, given its role in epigenetics. Compound 33 is an excellent start for further investigations.

 Question: To be extremely useful the drug should demonstrate pan-schistosomal cidal activity. Have the authors looked for similar lysine specific demethylases in other human schistosome species and determined their effectiveness?

Is the work clearly and accurately presented and does it cite the current literature?

Yes

If applicable, is the statistical analysis and its interpretation appropriate?

Yes

Are all the source data underlying the results available to ensure full reproducibility?

Yes

Is the study design appropriate and is the work technically sound?

Yes

Are the conclusions drawn adequately supported by the results?

Yes

Are sufficient details of methods and analysis provided to allow replication by others?

Yes

Reviewer Expertise:

Schistosome host-parasite interactions using a molecular, immunological and genetic approach.

I confirm that I have read this submission and believe that I have an appropriate level of expertise to confirm that it is of an acceptable scientific standard.

# Response

**Published:** 2023-06-16

**Authors:** Gilda Padalino

**Affiliations:** Cardiff University, UK

**We thank the reviewer very much for the detailed and constructive comments on our manuscript entitled: “** **Chemical modulation of Schistosoma mansoni lysine specific demethylase 1 (SmLSD1) induces wide-scale biological and epigenomic changes** **”** **, submitted to *Wellcome Open Research*.** Reviewer question: To be extremely useful the drug should demonstrate pan-schistosomal cidal activity. Have the authors looked for similar lysine specific demethylases in other human schistosome species and determined their effectiveness? We have explored the epigenetic enzymes involved in the methylation pathway in the other two major human infecting *Schistosome* species ( *S. haematobium* and *S. japonicum* – data unpublished). Briefly, the 14 *S. mansoni* Histone Demethylases (SmHDMs) homologous sequences (presented in a previous publication doi: https://doi.org/10.1016/j.ijpddr.2018.10.005) were used to identify the most closely related HDM homologues in *S. haematobium* and *S. japonicum*, respectively (Sh/SjHDMs). We used a BLASTp search within WormBase-Parasite using as threshold an E value of 1e ^-5^ or lower. As shown in **Figure 1**, we have identified at least a putative Lysine Specific Demethylase (LSD1) in *S. haematobium *(MS3 02208). Based on the stringent criteria set for this study, no close LSD1 homologue was identified in *S. japonicum*. We agree with the reviewer that exploring the biological role of this LSD1 homologue would be a valuable next step in progressing this epigenetic target (and its putative inhibitors), but we have yet to perform these experiments. However, the presence of homologous epigenetic enzymes in both *S. haematobium* and *S. japonicum* suggests the potential for developing a pan-schistosome drug capable of treating the three main species responsible for human schistosomiasis. 

FIGURE  

**Figure 1**. Schematic phylogeny of the HMTs and HDMs within the Schistosoma genus. Phylogenetic tree of *S. mansoni* HDMs and the closest S. haematobium or S. japonicum homologue is inferred from Maximum Likelihood analysis using MEGA. Bootstrap support values on the nodes range from 30-50 (highlighted with a circle), between 50 and 70 (highlighted with a square) and between 70 and 100 (highlighted with a star). Bootstrap support values below 30 are omitted. The phylogenetic tree is constructed with MEGA5 and the layout is modified with iTOL: Interactive Tree of Life (freely available on https://itol.embl.de/upload.cgi). Smp xxxxxx, Sjp xxxxxxx and MS3 xxxxx (with x representing any number) indicate respectively *S. mansoni*, *S. japonicum* and *S. haematobium* proteins.  

 We would like to thank the reviewer and the editor very much for the detailed and very constructive comments.

 Kind regards, Gilda Padalino (on behalf of the authors)
